# Prediction error is out of context: The dominance of contextual stability in structuring episodic memories

**DOI:** 10.3758/s13423-025-02723-4

**Published:** 2025-06-26

**Authors:** Berna Güler, Fatih Serin, Eren Günseli

**Affiliations:** 1 Department of Psychology, Sabancı University, Istanbul, Turkey; 2 MRC Cognition and Brain Sciences Unit, University of Cambridge, Cambridge, UK

**Keywords:** Event segmentation, Contextual stability, Prediction error, Episodic memory, Event segmentation theory

## Abstract

Our everyday experiences unfold continuously, yet we segment them into distinct memory units—a phenomenon known as event segmentation. Although extensively studied, the underlying mechanisms of event segmentation remain controversial. This study addresses this by comparing the two contrasting theories: prediction error and contextual stability. Across four experiments, we manipulated these factors separately to examine their distinct impacts on event segmentation, measured by temporal order and distance tasks. Experiments 1–3 demonstrate that contextual stability leads to more pronounced event segmentation than prediction errors in unstable contexts, underscoring its critical role. Experiment 4 further supported this by providing strong evidence for equally robust event segmentation for predicted and unpredicted transitions across stable contexts. We conclude that contextual stability plays a pivotal role in driving event segmentation, outweighing the effect of prediction errors. This study sheds new light on how our minds encode continuous experiences into coherent and meaningful memory units.

**Supplementary Information:**

The online version contains supplementary material available at 10.3758/s13423-025-02723-4.

## Introduction

Imagine walking on the beach and continuing your walk in the city. While the initial experience feels continuous, your memory will likely be segmented into distinct beach and city events. This process, known as *event segmentation*, involves parsing continuous experiences into discrete units in episodic memory (Zacks, 2019). It influences how we encode and remember experiences (Davachi & DuBrow, 2015; Heusser et al., 2018; Wu et al., 2023), making our memories easier to navigate (Michelmann et al., 2023).

Although the factors that generate distinct events are well studied, developing a global theory that involves these factors has been challenging. The dominant view is that event segmentation occurs due to *prediction errors.* According to the event segmentation theory, a prediction error signals the memory to create a new event (Rouhani et al., 2020; Zacks et al., 2009; Zacks & Swallow, 2007). Following a prediction error, the observer forms a representation of what is happening now, called an event model, to predict the future and compare this model to the ongoing experience (Bailey et al., 2013). This theory was supported by studies that found violations of predictions result in event segmentation, as seen in cases of unexpected changes in narratives, background colors, or object movements (Eisenberg et al., 2018; Heusser et al., 2018; Reynolds et al., 2007; Zacks, 2004; Zacks et al., 2011; Zacks & Tversky, 2001).

While event segmentation theory remains dominant, an alternative perspective, the *contextual stability* account, offers a different explanation. This account suggests that what drives segmentation is a transition across stable contexts, such as a change in object categories, task rules, or reward values (DuBrow & Davachi, 2016; Loh et al., 2016; Wang & Egner, 2022; Yates et al., 2023). Consistent with this, contextual overlap is critical for indices of segmented memories, such as coherent temporal encoding of events (Qiu et al., 2023; Schapiro et al., 2013; Sherman et al., 2023) and strong associations among items and sources (Polyn et al., 2009; Rouhani et al., 2023; Siefke et al., 2019). Moreover, an fMRI study showed that items within a context exhibit higher hippocampal pattern similarity than items across different contexts, with this enhanced similarity predicting smaller perceived temporal distances between within-context items, a key metric for segmented memories (Ezzyat & Davachi, 2014). Notably, even anticipated transitions and voluntary task switches, which are presumably free from prediction errors, have been shown to cause segmented memories (Pettijohn & Radvansky, 2016; Shim et al., 2024; Wang & Egner, 2022). Together, these studies suggest that having stable contexts, rather than experiencing prediction errors, is the main driver of segmentation.

Although these two views have been described in detail (Shin & DuBrow, 2021; Zacks et al., 2011; Zacks & Swallow, 2007), a conclusive comparison of their contributions to event segmentation has yet to be achieved. This gap may stem from the inherent challenge of dissociating these two views, as transitions in context have been suggested to produce prediction errors (Greve et al., 2017; Grisoni et al., 2021; Kim et al., 2014; Yazin et al., 2021). Consequently, effects typically attributed to prediction errors might also reflect shifts across stable contexts. For example, moving from one room to another while carrying virtual objects results in poorer memory performance for objects across rooms than objects from the same room (Radvansky & Copeland, 2006; Radvansky et al., 2011). This poorer performance can be interpreted in two ways: one perspective attributes it to prediction errors triggered by perceptual changes, while the other considers it the consequence of a shift from one stable context to another (Wang & Egner, 2022; Zacks et al., 2011).

Here, we developed an experimental procedure to overcome the challenge of disentangling the contributions of prediction errors and contextual changes in event segmentation by keeping prediction errors constant while manipulating contextual stability (Experiments 1–3) and keeping contextual stability constant while manipulating prediction errors (Experiment 4). Participants viewed images of real-life objects of various categories, and performed different task that provided different reward values (DuBrow & Davachi, 2016; Ezzyat & Davachi, 2014; Wang & Egner, 2022; Wen & Egner, 2022). In all experiments, violations of expectations regarding the upcoming object category, task rule, or reward value were considered prediction errors, such as when a series of a particular task rule was interrupted by another. Experiments 1-3 manipulated contextual stability by either having the same object category, task rule, or reward value across a series of successive items (prediction error + stable context) or having a deviant item interspersed across others (prediction error only). In Experiment 4, we kept contextual stability constant and manipulated prediction errors by providing a counter for the remaining number of items in an object category and task rule. Thus, a prediction error regarding a transition in object category or task rule was present only in the absence of a counter. In all experiments, after a brief intervening task that aimed to prevent rehearsal, participants completed temporal order and temporal distance tasks commonly used to assess event segmentation (Clewett et al., 2019; DuBrow & Davachi, 2013; Rouhani et al., 2020; Sols et al., 2017; Wang & Egner, 2022).

## Method

### Participants

Before conducting the study, we ran a statistical power analysis using Bayesian sequential design. We averaged the effect sizes across six studies that primarily measured event segmentation through temporal judgment tasks (D’Argembeau et al., 2015; Heusser et al., 2018; Horner et al., 2016; Sols et al., 2017; Van De Ven et al., 2022; Wang & Egner, 2022). We used an uninformed Cauchy distribution as the prior. For Experiments 1 and 2, the minimum and maximum number of participants were determined as 40 and 126, with a maximum power of .68 and a minimum power of .37 (false positive rate:.02), respectively. For Experiments 3 and 4, we ran the power analysis by considering the effect sizes of the within-subject design studies (Clewett et al., 2020; DuBrow & Davachi, 2013; Heusser et al., 2018; Horner et al., 2016; Sols et al., 2017; Van De Ven et al., 2022; Wang & Egner, 2022; Wen & Egner, 2022), and the minimum and maximum number of participants were determined as 53 and 240, with a maximum power of .57 and a minimum power of .40 (false positive rate:.02), respectively.

For Experiments 1 and 2, we ran 42 participants, and data from two participants were removed from further analyses due to being more than 2.5 standard deviations above or below the grand average in any of the measures. The main analyses were carried out with 40 participants in each experiment (Experiment 1: 29 women, *M*_age_ = 22.8 years, *SD*_age_ = 3.29; Experiment 2: 32 women, *M*_age_ = 22.5 years, *SD*_age_ = 2.21). For Experiment 3, we collected data from 63 participants. Data were removed from six participants due to the experiment crashing when participants pressed an invalid key, producing incomplete data and from four participants for being 2.5 standard deviations above or below the grand average in any of the measures. The analyses were conducted with 53 participants (29 men, *M*_*age*_ = 22.8 years, *SD*_*age*_ = 2.79). For Experiment 4, we initially collected data from 53 participants but did not observe event segmentation. To eliminate the possible explanation of fatigue, we conducted the same experiment with 12 rounds, as in previous studies (Heusser et al., 2018; Van De Ven et al., 2022) and collected data from 23 participants. During the data collection process, we spotted a mistake in the analysis code, the correction of which resulted in robust event segmentation both in the older 16-round version and the newer 12-round version (excluding two practice blocks from each condition). Therefore, we combined the data across these two versions, adding up to 76 participants in total. Six participants were removed for being 2.5 standard deviations above or below the grand average in any of the measures. The analyses were conducted with the remaining 70 participants (25 men, *M*_age_ = 22.2 years, *SD*_age_ = 2.76).

### Stimuli

A collection of 721 photographs of real-life objects, animals, and plants were used as memory items for Experiments 1 and 2, while a total of 910 animate and inanimate photographs were used for Experiments 3 and 4 (available in the OSF repository). We rescaled these photographs to have comparable pixel counts (500 × 500 pixels; 12.2° wide). The experiment was programmed in MATLAB via the Psychophysics Toolbox (Brainard, 1997; Pelli, 1997; Kleiner et al., 2007). The viewing distance was ~65 cm from the screen. The background was gray (RGB = [127.5 127.5 127.5]).

### Design

**Fig. 1:**
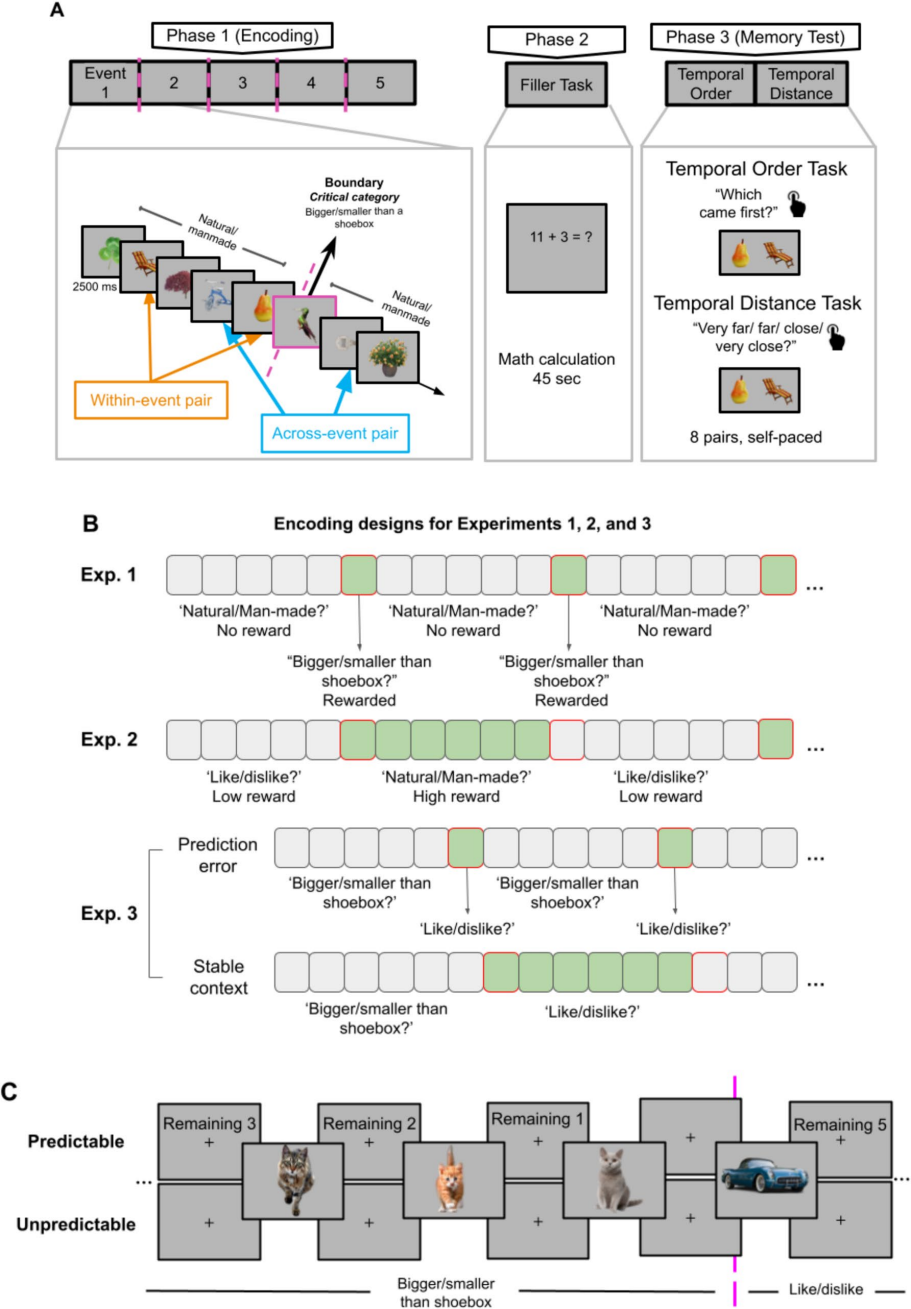
**A** Main phases of Experiment 1. There were three phases: encoding, filler task, and memory test. At the encoding phase, participants evaluated each image with a rule. In the memory test phase, participants performed temporal order and temporal distance judgment tasks for image pairs. **B** At the encoding phase, Experiment 1 aimed to create prediction errors by including a distinct object category, task rule, and reward value every 5–6 items. Experiment 2 involved stable contexts, where the transitioned task rule and reward value persisted for 5–6 items before another change. Experiment 3 manipulated these factors as a within-subject variable without reward. **C** Experiment 4 manipulated the predictability of event transitions with a counter for the remaining objects in a given category and task rule in half of the rounds. (Color figure online)

The experimental procedure for the experiments is shown in Fig. 1. Each round consisted of three phases: encoding, filler task, and memory test. The Experiments 1 and 2 took ~75 min, and Experiment 3 and 4 took ~90 min.

During encoding, participants evaluated the objects according to a task rule by using right or left arrow keys. Each response (e.g., like or dislike) was associated with one side of the screen and, therefore, one arrow key (i.e., left or right). Images did not repeat in the experiment and their assignment to conditions was counterbalanced across participants. The display duration for objects was 2,500 ms with a 2,000 ms interstimulus interval (ISI).

#### Experiment 1

Experiments 1 and 2 included 12 blocks, including one practice block. In Experiment 1, 30 items in each block came from three different object categories (real-life objects, animals, plants). One of the categories will be referred to as the critical category, and the other two as noncritical categories. Items from the critical category were evaluated with a different rule (e.g., bigger or smaller than a shoebox) than the noncritical categories (e.g., natural or manmade). Moreover, the critical category provided reward, while the noncritical categories provided no reward. The goal of switching the object category, task rule, and reward at “boundaries” was to increase the chances of segmentation. The critical category appeared in every six items (boundary items), and within-event items were from non-critical categories. Within-event image categories were equally chosen from noncritical categories and appeared in random order to ensure that each block had an equal number of noncritical categories. The task rule was shown on the screen during both the ISI and the object display. Images from the critical category were used to explore event boundaries. Participants were informed that they would evaluate the critical category items with a different rule and get a reward value for their judgments.

After encoding 30 images, participants performed basic mental summation for 45 s as a filler task (Wang & Egner, 2022). After the filler task, participants received the memory tests. During the memory test phase, participants were given eight image pairs that they encoded during the encoding phase. Half the pairs were within, and the other half were across-event items. The temporal distance was equal for each pair, as there were always two images between the tested pairs. For each image pair, participants first made temporal order judgments by indicating which item came first during encoding using left and right arrow keys corresponding to the pair items’ positions on the screen. The positions of the pair items were randomly shuffled for each tested pair. Next, participants made temporal distance judgments for the same image pair among four options (very far, far, close, very close) by assessing the temporal distance between images during encoding. Participants were also asked to indicate their level of confidence in their temporal order and temporal distance judgments (high or low confidence). Reaction time, confidence judgment, and accuracy were recorded.

#### Experiment 2

Experiment 2 was the same as Experiment 1, except for the following differences in Phase 1. First, participants evaluated every six consecutive items with the same rule (e.g., like/dislike) and the same reward value (e.g., high reward). In other words, task and reward values changed after every six items. Therefore, there was contextual stability during these items. Second, there was no critical category, as boundaries were determined via the stability of the task rule and reward value across six consecutive items.

#### Experiment 3

Experiment 3 was identical to Experiments 1 and 2 except for the following differences. The prediction error blocks (Experiment 1) and contextual stability blocks (Experiment 2) were combined into a single experiment as a within-subjects manipulation. There were 14 experimental blocks, seven for prediction error and seven for contextual stability. The block order was random except for avoiding repeating the same condition more than twice across consecutive blocks. Before the study, participants completed two practice rounds for each condition. All remaining changes aimed to simplify the design and reduce unnecessary variance in Phase 1. First, there was no reward manipulation. Second, each block had two predefined object categories (e.g., toys and flowers) instead of three, minimizing semantic relatedness and strengthening transitions. Third, instead of three rules, there were two rules throughout the experiment, which were “bigger or smaller than a shoebox” and “like or dislike.” Fourth, object categories, their presentation order within and across blocks, and task rules to evaluate each object category were counterbalanced across participants and conditions. Lastly, in prediction error blocks, each event consisted of five items from a single object category and were evaluated with one rule, and the boundary items were from the other category and were evaluated with the other rule. For contextual stability blocks, each event consisted of six items from a single object category and were evaluated with the same rule. Thus, for both conditions, the event transitions were every six items to ensure equal event onsets across conditions. This revision enabled the same order items to be asked in temporal order and temporal distance tasks in both conditions. In contrast, in Experiments 1 and 2, the offsets of events (i.e., the boundaries) were matched, not the onsets.

#### Experiment 4

Experiment 4 was identical to Experiment 3 except for the following changes that were introduced to manipulate prediction errors. There were two block types: In predictable blocks, a counter indicated how many items were left for the upcoming transition, which was displayed during ISI. There was no counter in unpredictable blocks (Fig. 1C). There were 16 blocks, though some participants completed only 12 blocks due to a coding mistake (see Participants). The first of each block type was for practice, leaving seven experimental blocks for each block type (and five for each block type in the 12-block version). The block type alternated every four blocks, and the order was randomized across participants. In each round of Phase 1, participants encoded 36 objects. Two changes were made to increase prediction errors. First, the object category repeated for either five, six, or seven objects, instead of being fixed. Second, for each run, each event length occurred twice in a run in a pseudorandom order, without repetition across consecutive events (Shim et al., 2024). On the memory test (Phase 3), there were six item pairs: three within events and three across events.

## Results

Analyses were conducted with JAMOVI 2.3 (2023) and the Bayesian Methods module (jsq). Bayesian paired and independent-samples *t* tests compared the within-subject differences for within versus across boundary effects. Confidence judgments and RTs for each measure were reported in the Supplementary Material. BF_10/01_ values below 1 were considered as no evidence, 1–3 as anecdotal evidence, 3–6 as moderate evidence, 6–10 as substantial evidence, and larger than 10 as strong evidence for H1/H0.

## Experiment 1: Does prediction error generate event boundaries?

**Fig. 2:**
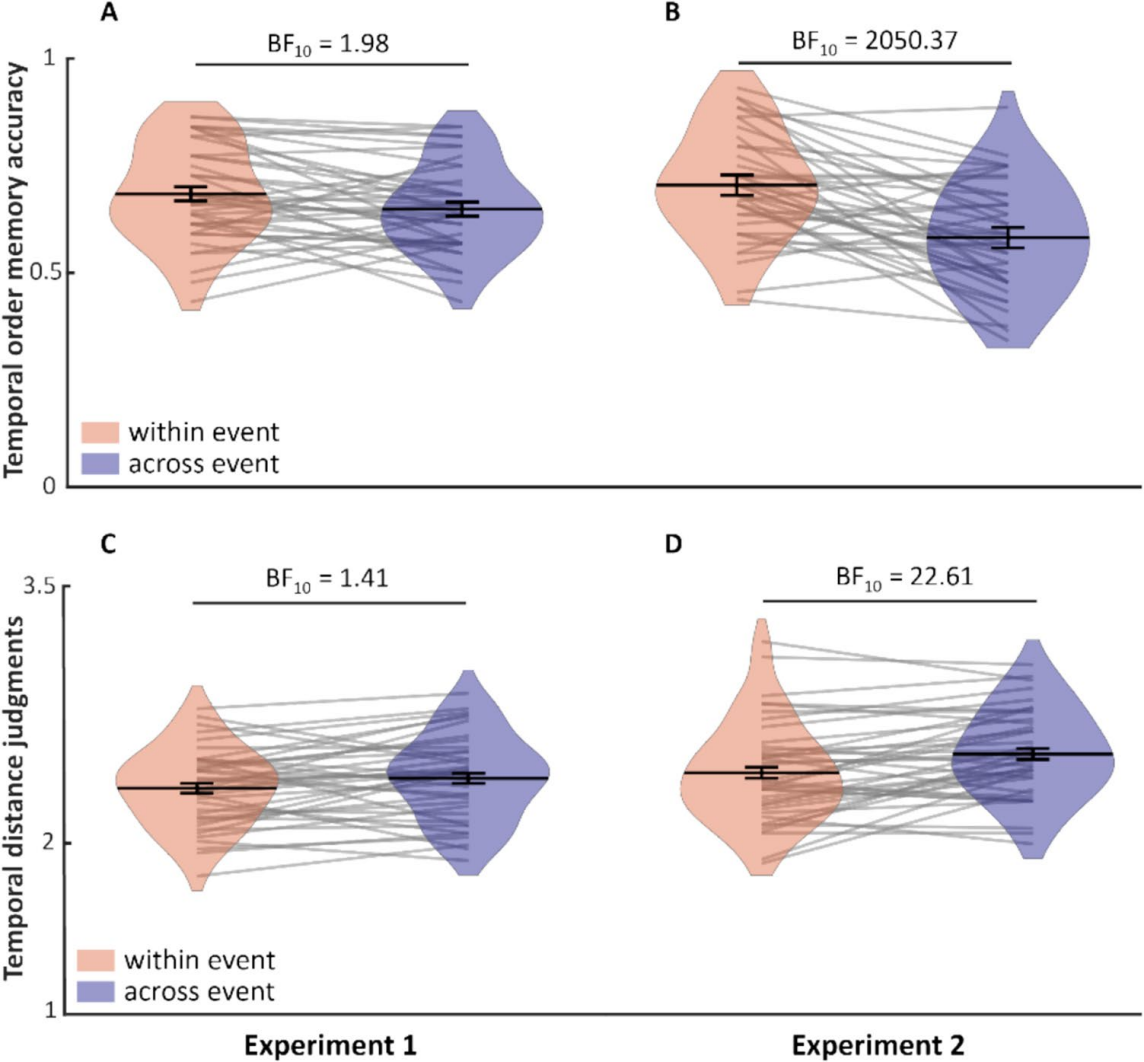
**A–B** Temporal order accuracy and (**C–D**) temporal distance judgments for within-event and across-event items for Experiment 1 and Experiment 2. (Color figure online)

For temporal order memory, accuracy was higher for within-event pairs (*M* =.68, *SD* =.11) than across-event pairs (*M* =.64, *SD* =.10), with anecdotal evidence, BF_10_ = 1.98 (Fig. 2A). For temporal distance judgments, reported distance was higher for across-event pairs (*M* = 2.41, *SD* =.24) than within-event pairs (*M* = 2.35, *SD* =.22), also with anecdotal evidence, BF_10_ = 1.41 (Fig. 2C). Thus, Experiment 1 provided only anecdotal evidence for event segmentation, as reflected by higher temporal order accuracy and shorter perceived distance for within versus across events.

## Experiment 2: Does contextual stability generate event boundaries?

For temporal order memory, accuracy was higher for within-event (*M* =.70, *SD* =.12) compared with across-event pairs (*M* =.58, *SD* =.12), with strong evidence, BF_10_ = 2050.37 (Fig. 2B). For temporal distance judgments, temporal distance was higher for across-event pairs (*M* = 2.43, *SD* =.25) than within-event pairs (*M* = 2.32, *SD* =.29), with strong evidence, BF_10_ = 22.61 (Fig. 2D). Thus, in Experiment 2, we obtained strong evidence for event segmentation.

### Between-subjects comparison across Experiments 1 and 2

To evaluate the contributions of contextual stability and prediction error to event segmentation, we performed between-subjects *t* tests on segmentation scores across Experiments 1 and 2. Segmentation scores were calculated by subtracting the mean accuracy for across-event pairs from within-event pairs for the temporal order task. For the temporal distance task, the subtraction was reversed so that higher values consistently indicated larger segmentation. This procedure was applied to accuracy, RT, and confidence judgments for both tasks and across experiments.

**Fig. 3:**
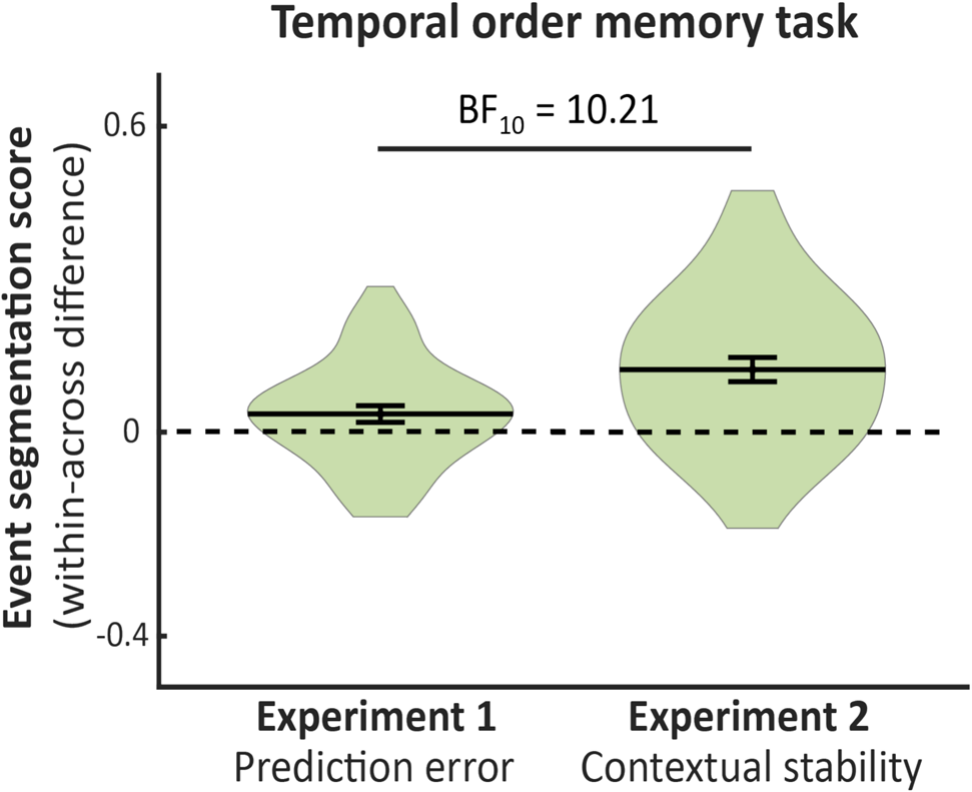
Temporal order memory accuracy difference between within and across items for Experiments 1 and 2

The segmentation score for temporal order accuracy was higher in Experiment 2 (*M* =.12, *SD* =.11) than in Experiment 1 (*M* =.03, *SD* =.10), with strong evidence, BF_10_ = 10.21. The segmentation score for temporal distance did not differ between Experiment 1 (*M* = −.05, *SD* =.18) and Experiment 2 (*M* = −.10, *SD* =.20), with anecdotal evidence for the null, BF_01_ = 1.52. These results suggest that contextual stability is more important than prediction errors in generating event boundaries Fig. 3.

## Experiment 3: Within-subject replication of Experiments 1 and 2

In prediction error blocks, temporal order memory accuracy for within-event pairs (*M* =.64, *SD* =.11) was higher than across-event pairs (*M* =.57, *SD* =.07), with strong evidence, BF_10_ = 92.4. Similarly, in contextual stability blocks, temporal order memory accuracy was higher for within-event pairs (*M* =.73, *SD* =.12) than for across-event pairs (*M* =.60, *SD* =.11), with strong evidence, BF_10_ = 2.08e+7.

For temporal distance judgments in the prediction error blocks, there was no difference between within (*M* = 2.40, *SD* =.30) and across-event pairs (*M* = 2.43, *SD* =.29), with moderate evidence, BF_01_ = 4. In contrast, in contextual stability blocks, temporal distance was evaluated higher for across-event pairs (*M* = 2.40, *SD* =.31) than within-event pairs (*M* = 2.18, *SD* =.32), with strong evidence, BF_10_ = 436286.

**Fig. 4:**
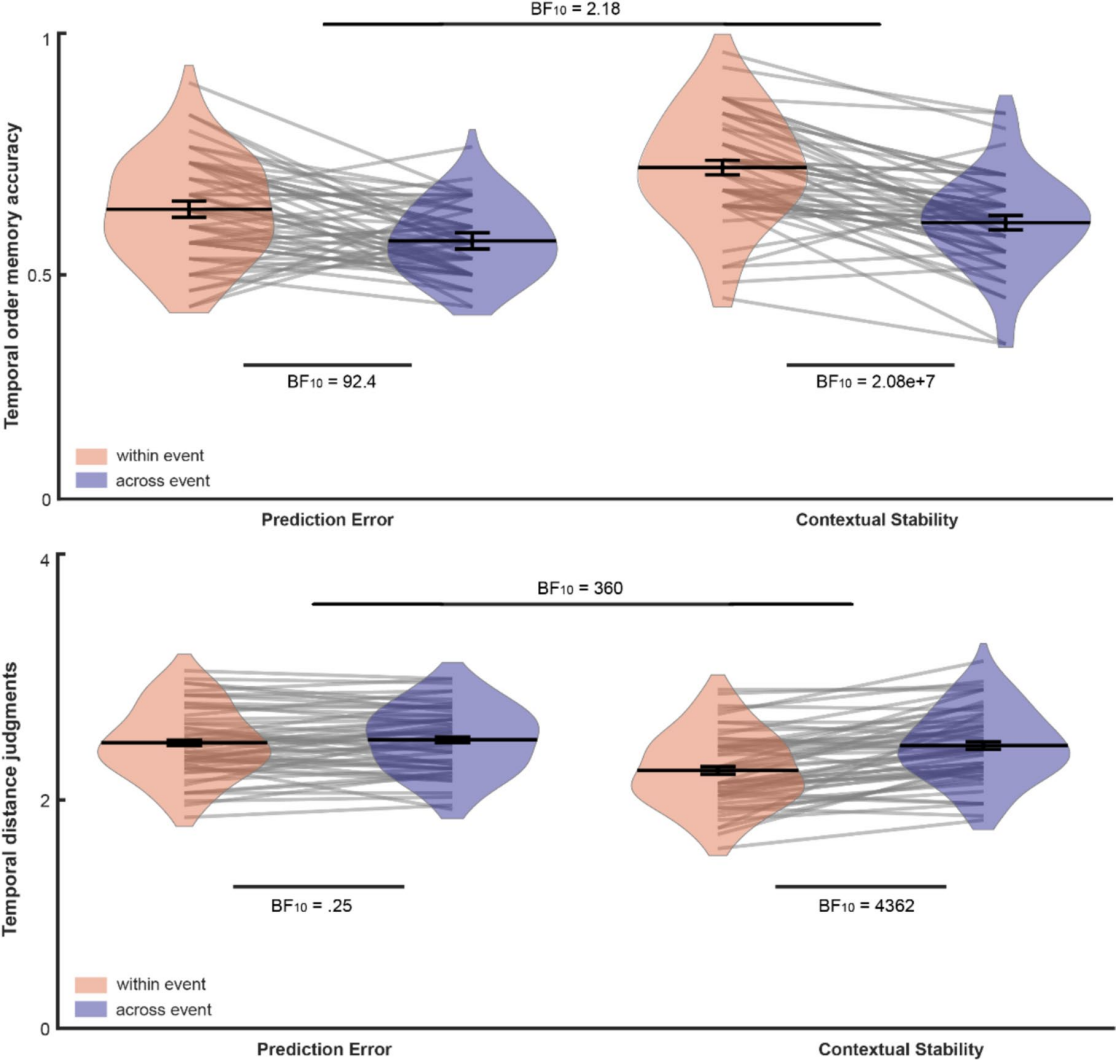
Temporal order memory and temporal distance judgments for Experiment 3. The Bayes factor above reflects the comparison of the segmentation scores (within-across difference) across conditions. (Color figure online)

To directly compare segmentation across conditions, we computed the difference scores as segmentation indices. For temporal order, segmentation was higher for contextual stability (*M* =.12, *SD* =.12) than for prediction errors (*M* =.07, *SD* =.13), with anecdotal evidence, BF_10_ = 2.18. For temporal distance judgments, segmentation was higher for contextual stability (*M* =.21, *SD* =.24) than prediction errors (*M* =.02, *SD* =.17), with strong evidence, BF_10_ = 360 (Fig. 4).

## Experiment 4: Does prediction error play a role?

In predictable blocks, temporal order accuracy was higher for within-event pairs (*M* =.68, *SD* =.13) than for across-event pairs (*M* =.56, *SD* =.10), with strong evidence, BF_10_ = 1.53e+6. Similarly, in unpredictable blocks, temporal order accuracy was higher for within-event pairs (*M* =.67, *SD* =.13) than for across-event pairs (*M* =.56, *SD* =.11), with strong evidence, BF_10_ = 135011.

Temporal distance judgments also showed strong segmentation effects in both conditions: In predictable blocks, the evaluated distance was higher for across-event pairs (*M* = 2.57, *SD* =.33) than for within-event pairs (*M* = 2.24, *SD* =.34), with strong evidence, BF_10_ = 7.93e+7. Similarly, in unpredictable blocks, the evaluated distance was higher for across-event pairs (*M* = 2.54, *SD* =.32) than within-event pairs (*M* = 2.26, *SD* =.31), with strong evidence, BF_10_ = 1.27e+8.

**Fig. 5:**
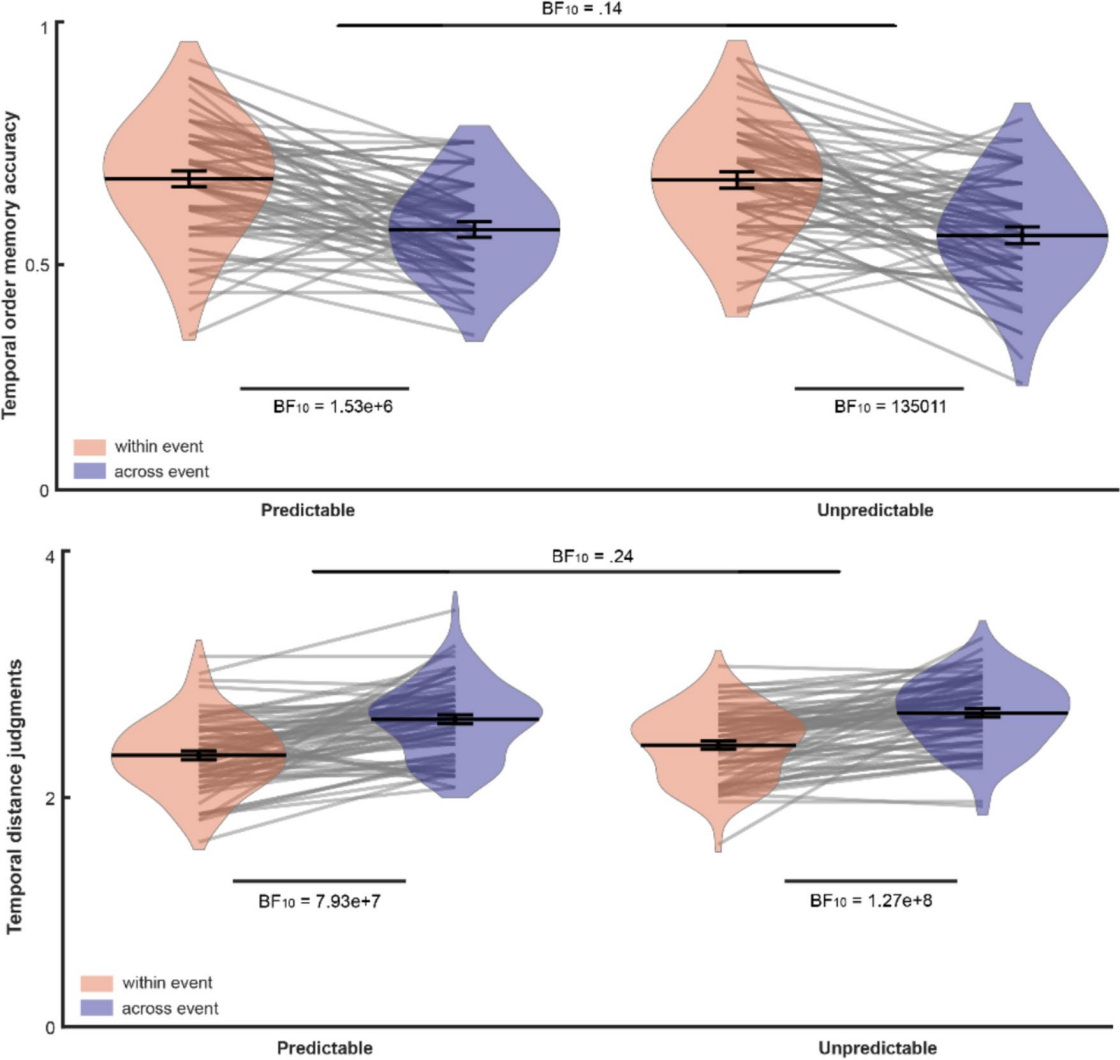
Temporal order memory and temporal distance judgments for Experiment 4. The Bayes factor above reflects the comparison of the segmentation scores (within-across difference) across conditions. (Color figure online)

To directly compare segmentation across conditions, we computed the difference scores for each measure. For temporal order memory, segmentation did not differ between predictable (*M* =.12, *SD* =.15) and unpredictable blocks (*M* =.11, *SD* =.16), with substantial evidence for the null, BF_01_ = 7.12. Similarly, for temporal distance, segmentation did not differ between predictable (*M* =.33, *SD* =.36) and unpredictable blocks (*M* =.28, *SD* =.30) with moderate evidence for the null, BF_01_ = 4.02 (Fig. 5). Thus, there was equal segmentation across predictable and unpredictable blocks.

## Discussion

We contrasted two accounts of the underlying mechanism of event segmentation. The prevailing view in the literature posits that event boundaries are shaped by prediction errors (Zacks, 2004; Zacks et al., 2007). In contrast, an alternative theory proposes that transitions across stable contexts drive event boundaries (Shin & DuBrow, 2021). A direct comparison of the two views has been challenging due to the difficulty of disentangling their respective influences. Here, we overcame this challenge by independently manipulating each factor across four experiments.

In Experiment 1, we introduced a transient change in object, rule, and reward categories to induce prediction errors, while in Experiment 2, the changed task rule and reward persisted for 5–6 more items to establish contextual stability. Segmentation was larger in Experiment 2 than in Experiment 1. We replicated this result in Experiment 3 using a within-subjects design. Observing more robust event segmentation when events have more consistent contextual information emphasizes the importance of contextual stability. However, it can be argued that contextual stability leads to higher prediction errors and, hence, better event segmentation. To tackle this, in Experiment 4, we manipulated prediction errors by providing a counter for the remaining number of items before transitions across stable contexts. Segmentation was equally robust for predictable and unpredictable transitions, revealing no effect of prediction errors. These outcomes challenge the influential event segmentation theory and instead emphasize the pivotal role of contextual stability in event segmentation.

Various aspects of contextual stability can segment events. First, having a common context can contribute to the structuring of memories by promoting associations among information within the same context and improving sequential order memory (Davachi & DuBrow, 2015; DuBrow & Davachi, 2016; Ezzyat & Davachi, 2011; Farrell, 2012; Horner et al., 2016; Pu et al., 2022; Rouhani et al., 2020; Sols et al., 2017). These associations may contribute to the segmented structure during retrieval, given the importance of context binding for episodic memory retrieval (Ezzyat & Davachi, 2011; Yonelinas et al., 2019). Such associations can also assist in relating the current event with remote episodic memories that share the same context, helping to generalize from similar experiences (Hahamy et al., 2023). Second, contextual shifts may drive the removal of prior goals and task-related information from memory and their update with information appropriate for the new context. In line with this, we recently demonstrated that a context change triggers the reactivation of task-related long-term memories in working memory (Özdemir et al., 2024). This reactivation might explain how updating task-related information occurs at event boundaries.

As the field advances, it becomes crucial to clarify both the mechanisms behind segmentation and the ways different memory measures capture its components. Although prediction errors have long been considered central to event segmentation, previous findings may reflect transitions across stable contexts, as prediction errors occur at transitions in perception, reward, or semantics (Rouhani et al., 2020; Zacks, 2004). Given the overlap of contextual shifts and prediction errors during encoding, future studies should exercise caution while interpreting the contributions of each to memory structuring. An open question for future research is to reveal which of the two is critical for triggering an event boundary: the contextual change itself or its stability after a transition.

Moreover, identifying what drives segmentation is closely tied to how it is measured, as different memory tasks may capture distinct facets of the same underlying process. We found the segmentation difference for temporal order memory between Experiments 1 and 2, while the condition difference was more robust for temporal distance judgments in Experiment 3. These differences across experiments may be attributable to noise, or they may also reflect different underlying memory processes tapped by the two tasks (DuBrow & Davachi, 2014; Pu et al., 2022; Wang & Egner, 2022), which can be evaluated by future studies.

Despite the contributions of the current study, two limitations can be addressed. First, in Experiment 4, participants may have noticed that transitions consistently occurred every five to seven items in the unpredictable condition. This regularity could have provided some predictability, thereby reducing the degree of prediction errors (Greve et al., 2017, 2019; Van Kesteren et al., 2012). Such a reduction may partly explain the absence of segmentation difference between conditions. Nevertheless, the presence of strong event segmentation in the predictable condition, where prediction errors were minimized, provides compelling evidence that contextual stability is the primary factor for segmentation. Second, while our findings suggest that prediction errors, as operationalized in our paradigm, are not sufficient to induce segmentation without contextual stability, different types of prediction errors may still contribute to segmentation. For example, a recent study demonstrated that a single highly negative arousing image embedded within a sequence of neutral stimuli was sufficient to trigger segmentation (Harris et al., 2025). Thus, strong prediction errors involving survival-related information, such as threat signals, may be sufficient to segment episodic memories. However, since the arousing stimulus occurred in a fixed, predictable position, segmentation in that study may have been driven by arousal itself rather than by prediction error per se.

We would like to clarify that we are not arguing more broadly against the influence of prediction errors on memory. Although our findings show that prediction errors are insufficient to drive event segmentation in the absence of stable contexts, prediction errors play a well-established role in various memory-related processes (Loock et al., 2025; Ortiz-Tudela et al., 2024; Pupillo et al., 2023). For example, prediction errors have been shown to enhance memory encoding without generating segmentation (Wang & Egner, 2023) by increasing attention to unexpected stimuli (Greve et al., 2017), and to support memory updating by facilitating the integration of new information that violates prior expectations (Sinclair et al., 2021). Additionally, prediction errors can trigger memory reorganization, restructuring the temporal or semantic relationships among events (Bein et al., 2020; Rouhani et al., 2020; Swallow et al., 2022).

To conclude, we found that switching across stable contexts contributes to event segmentation above and beyond prediction errors. The lack of event segmentation when only prediction error but not contextual stability was present challenges the prevailing event segmentation theory that posits segmentation is driven by prediction errors. These findings underscore the need for a comprehensive understanding of event segmentation mechanisms, shedding light on how our minds organize and remember our experiences.

## Supplementary Information

Below is the link to the electronic supplementary material.

Supplementary file1 (DOCX 3091 KB)

## Data Availability

Analysis script and raw data files are available on the Open Science Framework repository (https://osf.io/zasmy/).

## Code availability

Codes for the experiments are available on the Open Science Framework repository (https://osf.io/zasmy/).
