# Supplementary material for: Prediction error is out of context: The dominance of contextual stability in structuring episodic memories
Source: Psychon Bull Rev. 2025 Jun 26;32(6):2957–68. doi: 10.3758/s13423-025-02723-4 (PMC12627157; doi:10.3758/s13423-025-02723-4)
Supplement: Supplementary file 1 — Supplementary file1 (DOCX 3091 KB) [file 13423_2025_2723_MOESM1_ESM.docx]

**Supplemental Material**

**Results**

Response times (RT) and confidence judgments were provided in the supplementary materials due to space constraints.

**Experiment 1**

Confidence judgments for temporal order judgments were lower for across-event pairs (*M* = .65, *SD* = .18) than within-event pairs (*M* = .69, *SD* = .16), BF_10_ = 46.18. RT in order judgments were higher for across-event pairs (*M* = 3.49, *SD* = 1.35) compared to within-event pairs (*M* = 3.38, *SD* = 1.23), with anecdotal evidence, BF_10_ = 1.80 (Supplementary Figure 1A & 2A). There was anecdotal evidence for equal temporal distance judgment RT for within-event (*M* = 1.48, *SD* = .38) and across-event pairs (*M* = 1.45, *SD* = .34), BF_01_ = 1.54 (Supplementary Figure 3A). Moreover, there was moderate evidence for indifference in temporal distance confidence judgments between within-event pairs (*M* = .70, *SD* = .26) and across-event pairs between item pairs (*M* = .67, *SD* = .26), BF_01_ = 3.11 (Supplementary Figure 4A).

**Experiment 2**

Confidence judgments for order judgments were higher for within-event (*M* = .69, *SD* = .15) compared to across-event pairs (*M* = .66, *SD* = .14), with anecdotal evidence, BF_10_ = 1.75 (Supplementary Figure 2B). RTs during order judgments were higher for across-event pairs (*M* = 3.98, *SD* = 1.46) compared to within-event pairs (*M* = 3.70, *SD* = 1.40), BF_10_ = 60.55 (Supplementary Figure 1B). Confidence judgments for temporal distance were higher for within-event pairs (*M* = .72, *SD* = .18) than across-event pairs (*M* = .69, *SD* = .21), with anecdotal evidence, BF_10_= 1.19 (Supplementary Figure 4B). There was substantial evidence for the indifference of RTs for temporal distance judgments for within (*M* = 1.65, *SD* = .50) and across-event pairs (*M* = 1.66, *SD* = .48), BF_01_ = 3.14 (Supplementary Figure 3B).

**Comparison between Experiments 1 & 2**

For the comparison between Experiment 1 and 2, the event segmentation score (within- minus across-event scores) for temporal order RTs was higher for Experiment 2 (*M* = .27, *SD* = 45) than for Experiment 1 (*M* = .11, *SD* = .32), with anecdotal evidence, BF_10_ = 1.38. There was indifference between experiments for confidence judgments for temporal order (BF_01_ = 2.35), temporal distance RTs (BF_01_ = 1.92), and confidence judgments for temporal distance judgments (BF_01_ = 2.40), with anecdotal evidence.

**Experiment 3**

The measures were evaluated for the prediction error blocks. There was no evidence for the difference between within and across-pairs for RT in temporal order memory (BF_10_ = 0.15), confidence judgments in temporal order memory (BF_10_ = 0.22), RTs for temporal distance judgments (BF_10_ = 0.15), and confidence judgments for temporal distance judgments (BF_10_ = 0.38).

For the contextual stability blocks, RTs in temporal order memory were higher for across-event pairs (*M* = 4.61, *SD* = 4.16) than within-event pairs (*M* = 3.85, *SD* = 2.42), BF_10_ = 3.95. Confidence judgments for within-events (*M* = .73, *SD* = .15) were higher than across-event pairs (*M* = .64, *SD* = .18) in temporal order with strong evidence, BF_10_ = 567.31. Confidence judgments for temporal distance judgments were higher for within-events (*M* = .73, *SD* = .23) than across-events (*M* = .67, *SD* = .25), with strong evidence, BF_10_ = 23.58. There was no evidence for the difference between across and within-event pairs’ RTs for temporal distance judgments (BF_10_ = .41).

We compared the difference scores for each measure between conditions. RTs for temporal order indicated higher segmentation in contextual stability (*M* = .76, *SD* = 2.07) than prediction error blocks (*M* = .02, *SD* = 1.60), with anecdotal evidence, BF_10_ = 2.26. Confidence judgments for temporal order generated higher segmentation for contextual stability (*M* = .08, *SD* = .13) than prediction error (*M* = .01, *SD* = .10), with substantial evidence, BF_10_ = 7.02. RTs (BF_10_ = .22) and confidence judgments (BF_10_ = 0.60) for temporal distance judgments were not differentiated between conditions.

**Experiment 4**

For the predictable blocks, RTs were higher during within-event (*M* = 3.16, *SD* = 1.00) than across-event pairs (*M* = 3.53, *SD* = 1.38) for temporal order memory, BF_10_ = 151.90. Confidence judgments for within-event pairs (*M* = .68, *SD* = .19) were higher than across-event pairs (*M* = .60, *SD* = .21) for temporal order memory, BF_10_ = 192.04. Similarly, confidence judgments for within-event pairs (*M* = .69, *SD* = .25) were higher than across-event pairs (*M* = .63, *SD* = .29) for temporal distance memory, BF_10_ = 113.09. There was no evidence for the difference between within and across-event pairs for RTs for temporal distance judgments (BF_10_ = .16).

For the unpredictable blocks, RTs were higher for across (*M* = 3.63, *SD* = 1.51) than within-event pairs (*M* = 3.35, *SD* = 1.23) for temporal order memory, BF_10_ = 4.05. Confidence judgments were higher for within-event pairs (*M* = .69, *SD* = .19) than across-event pairs (*M* = .57, *SD* = .21) for temporal order memory, BF_10_ = 159250. Similarly, confidence judgments were higher for within-event pairs (*M* = .69, *SD* = .24) than across-event pairs (*M* = .64, *SD* = .27) for temporal distance judgments, BF_10_ = 32.82. There was no evidence for the difference between within and across-event pairs for RTs for temporal distance judgments (BF_10_ = .20)

We compared the difference scores for each measure between conditions. There was no evidence for the difference in segmentation scores between variables (BF_10_ ≥ .13).


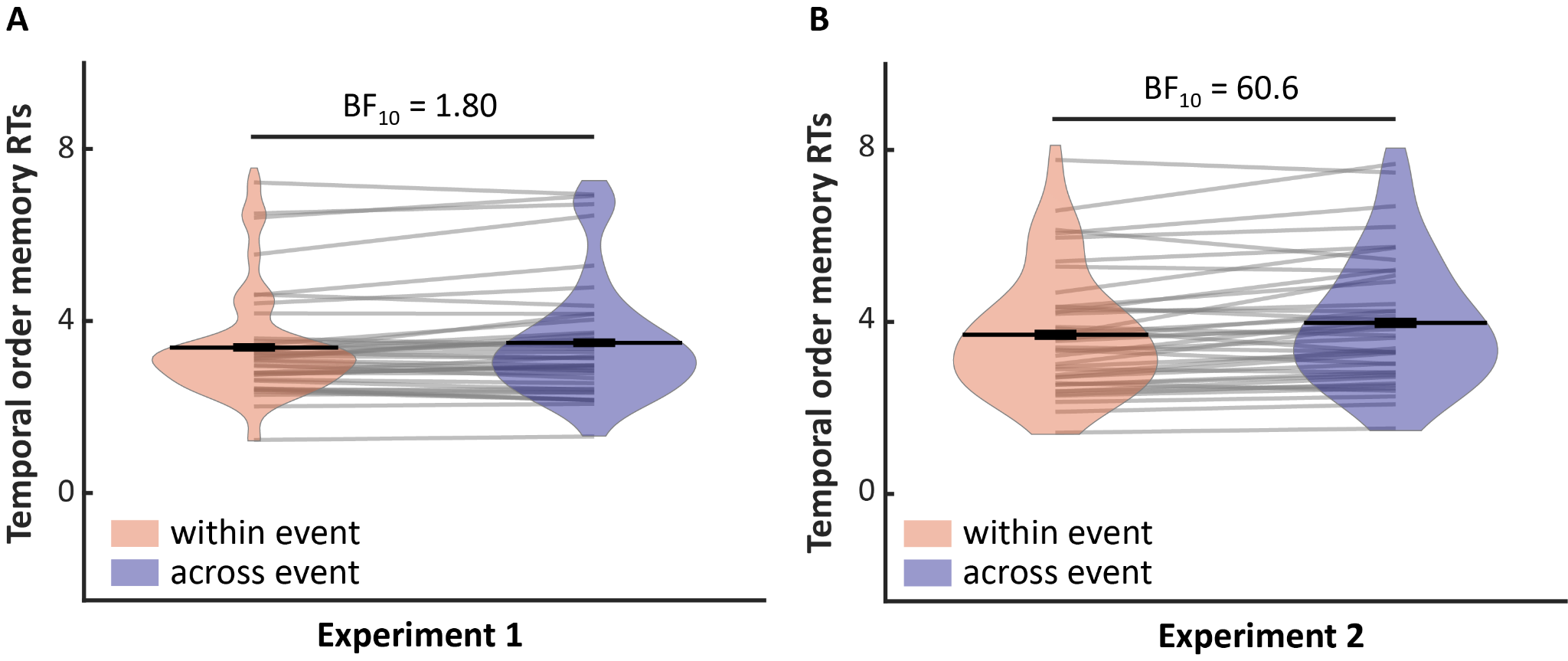


**Supplementary Figure 1**. Temporal order memory RTs for within and across-event pairs in Experiment 1 and Experiment 2.


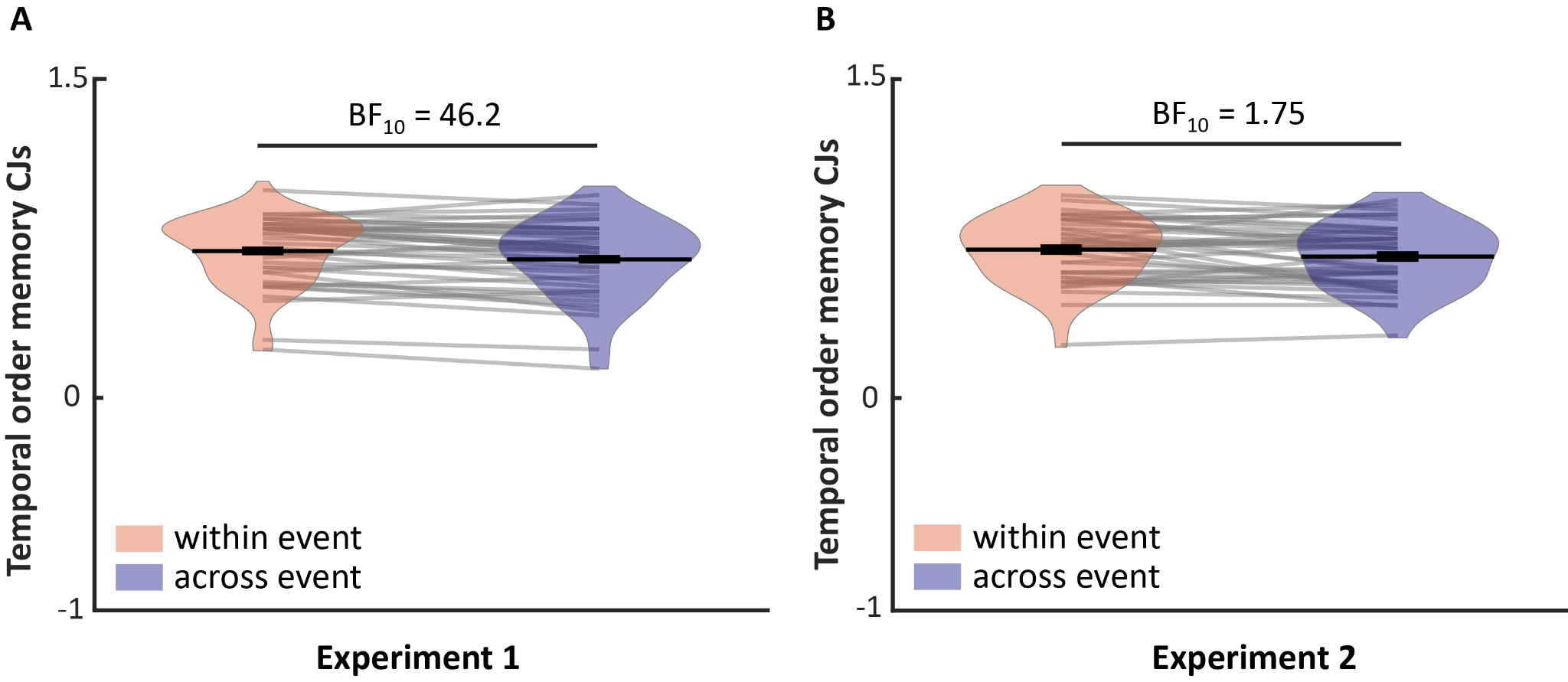


**Supplementary Figure 2**. Temporal order memory confidence judgments for within and across-event pairs in Experiment 1 and Experiment 2.


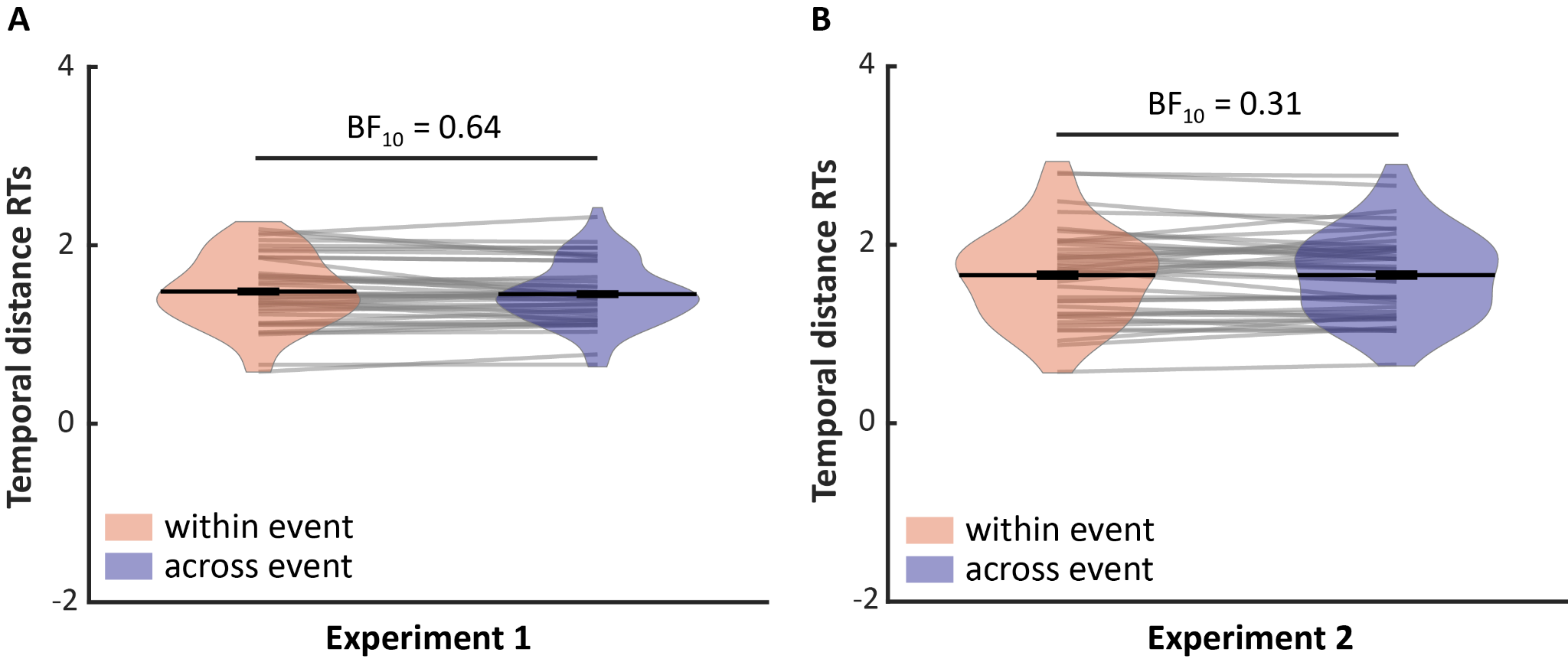


**Supplementary Figure 3**. RTs for temporal distance judgments for within and across-event pairs in Experiment 1 and Experiment 2.


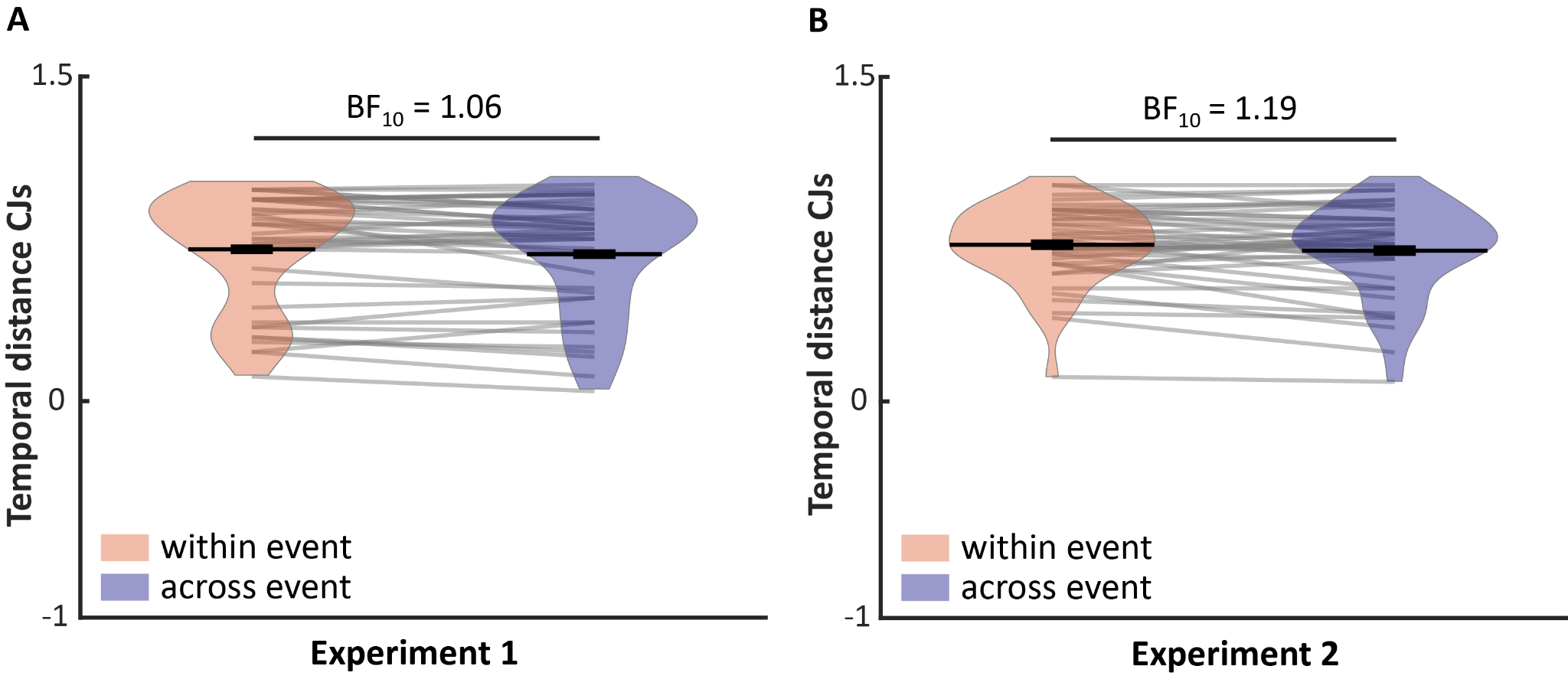


**Supplementary Figure 4.** Confidence judgments for temporal distance judgments for within and across-event pairs in Experiment 1 and Experiment 2.


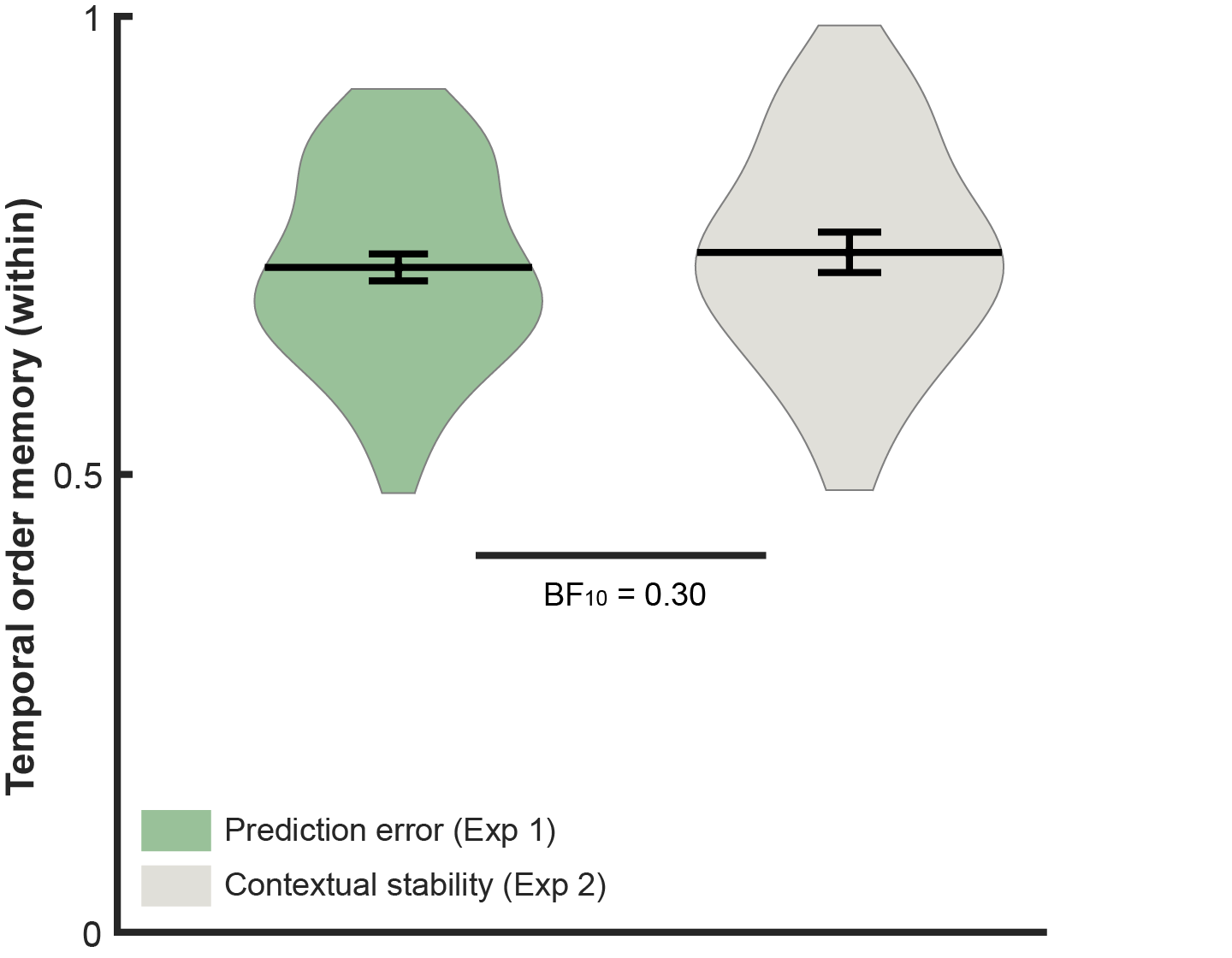

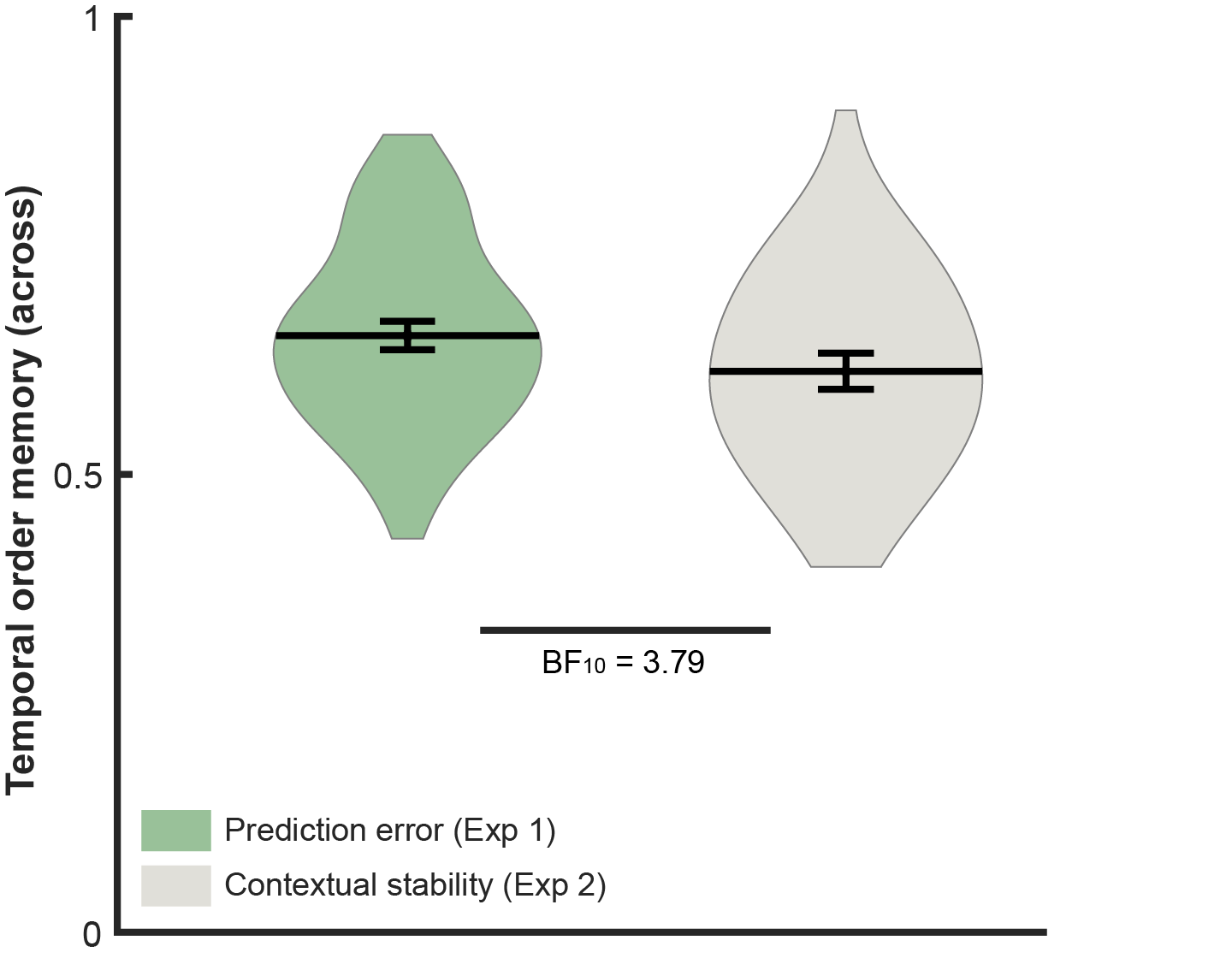


**Supplementary Figure 5.** Temporal order memory accuracy for within (left) and across-event pairs (left) between Experiments 1 and 2.


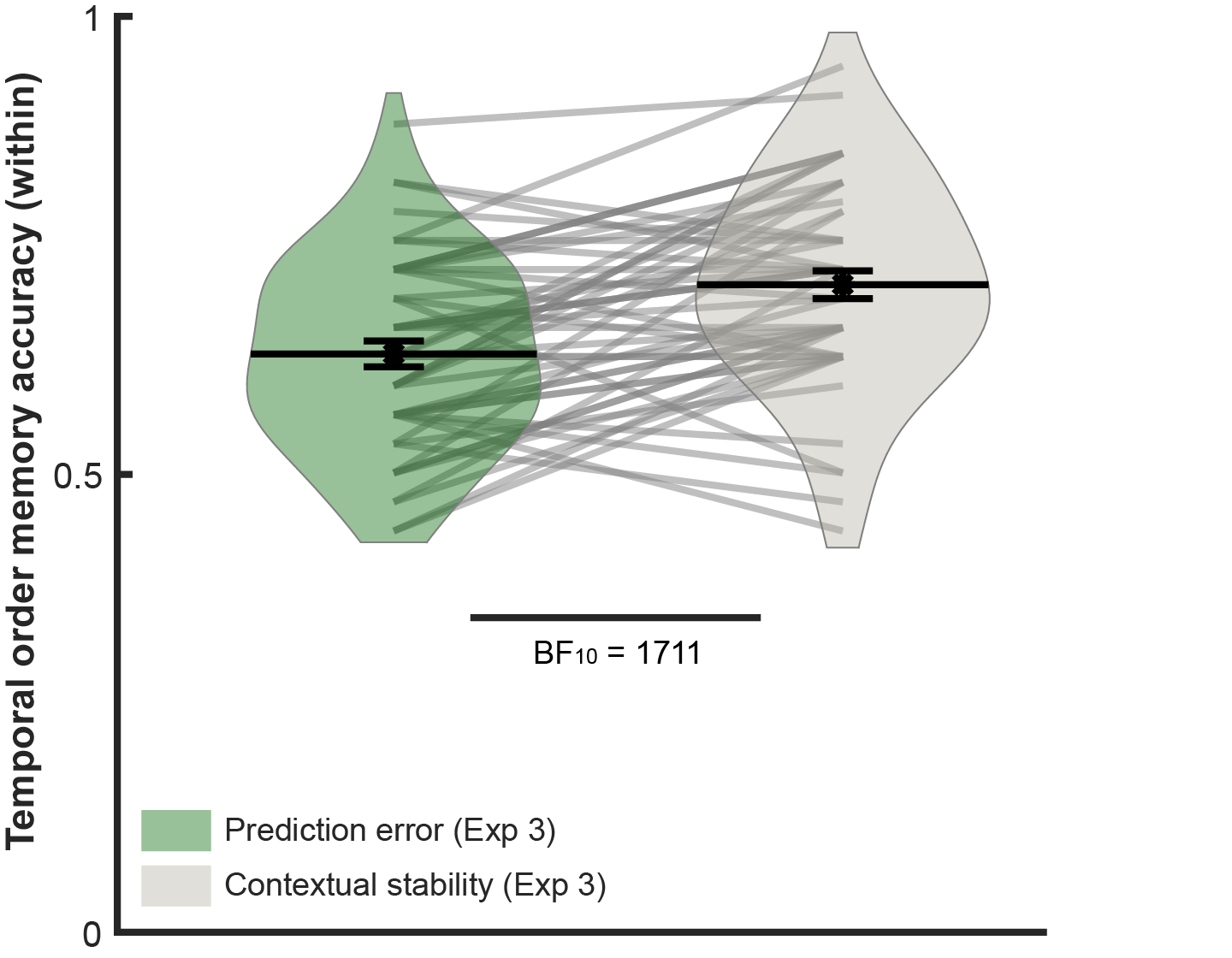

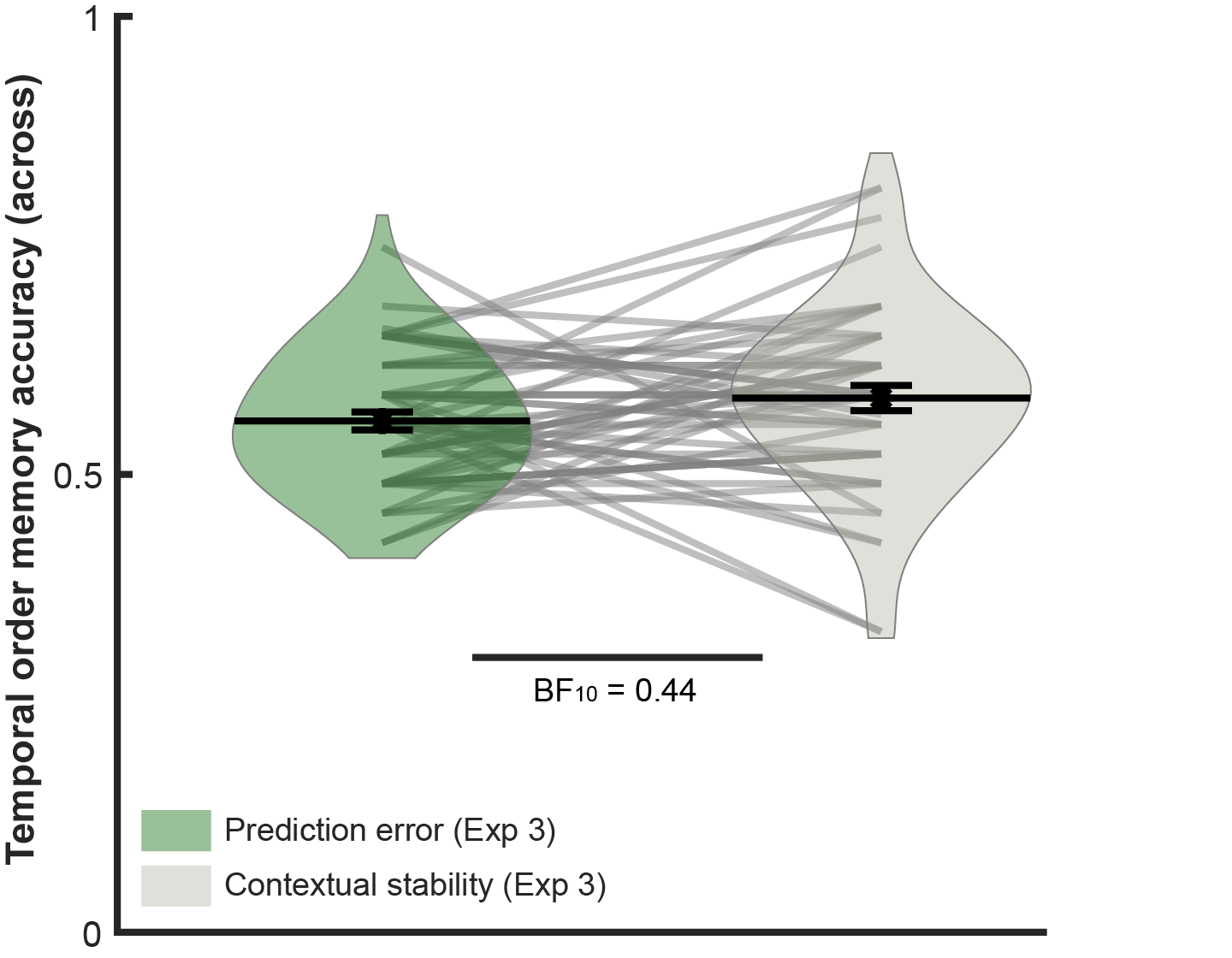


**Supplementary Figure 6.** Temporal order memory accuracy for within (left) and across-event pairs (right) between conditions for Experiment 3.


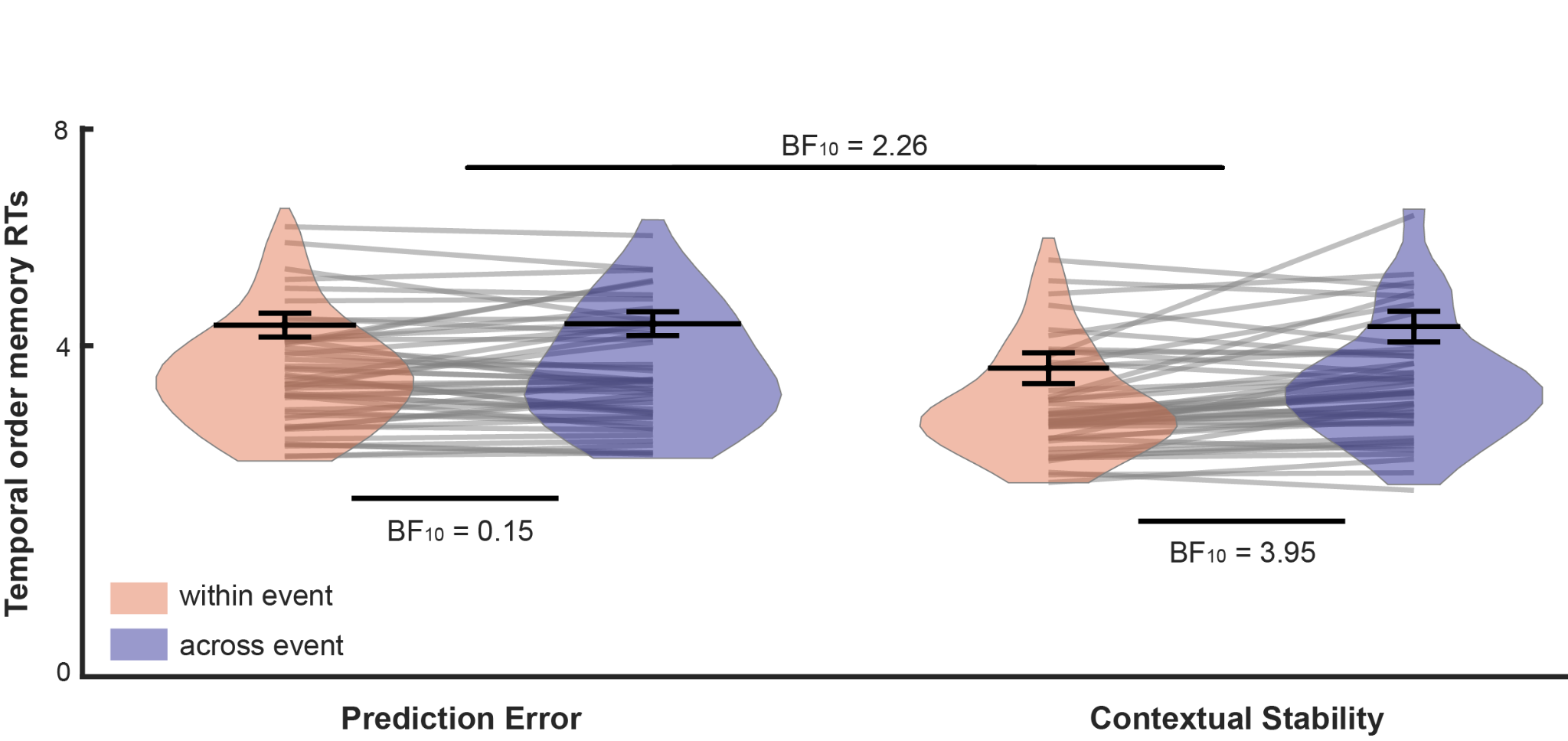


**Supplementary Figure 7.** Temporal order memory RTs for prediction error and contextual stability blocks in Experiment 3. The Bayes Factor above reflects the comparison of the segmentation scores (within-across difference) across conditions.


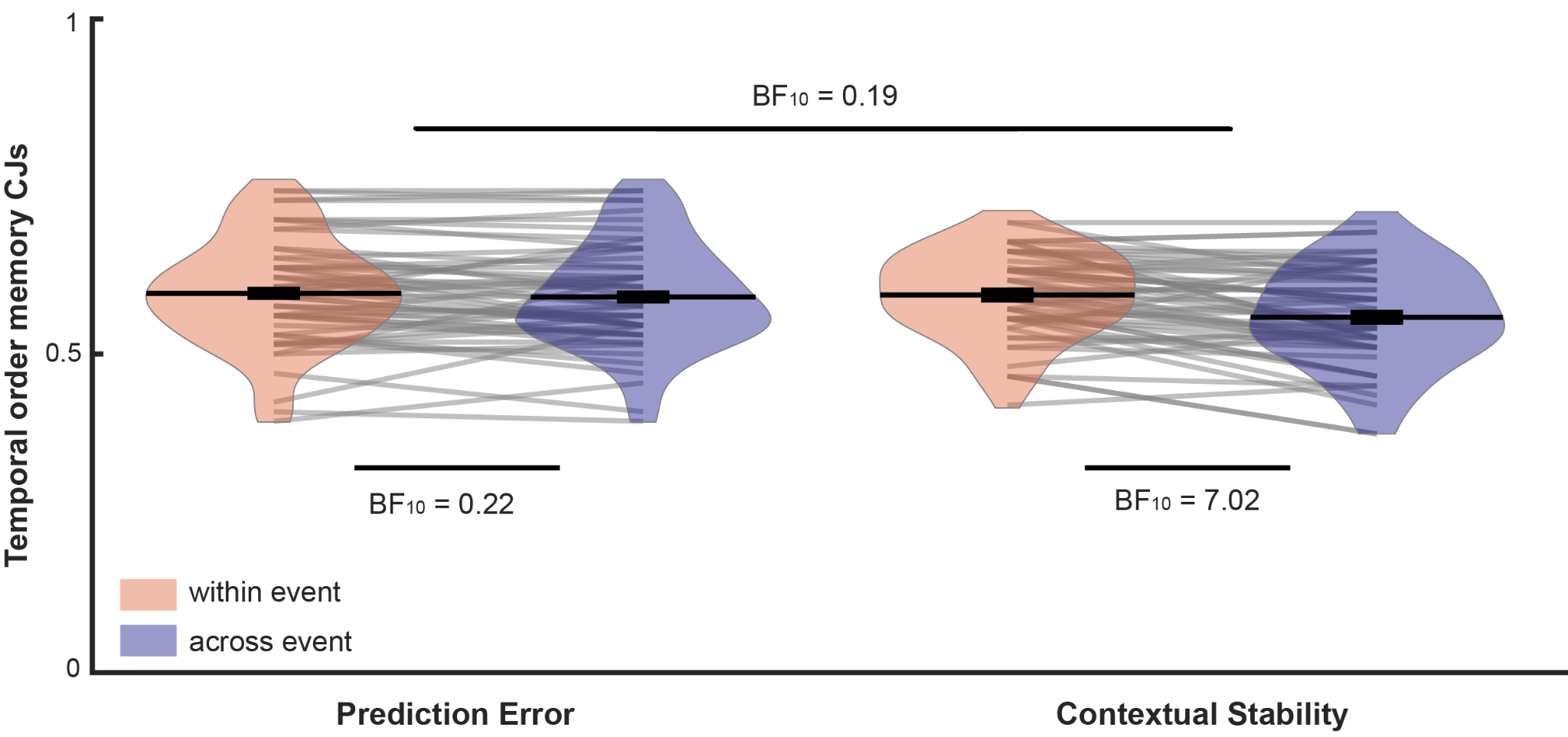


**Supplementary Figure 8.** Temporal order memory confidence judgments for prediction error and contextual stability blocks in Experiment 3. The Bayes Factor above reflects the comparison of the segmentation scores (within-across difference) across conditions.


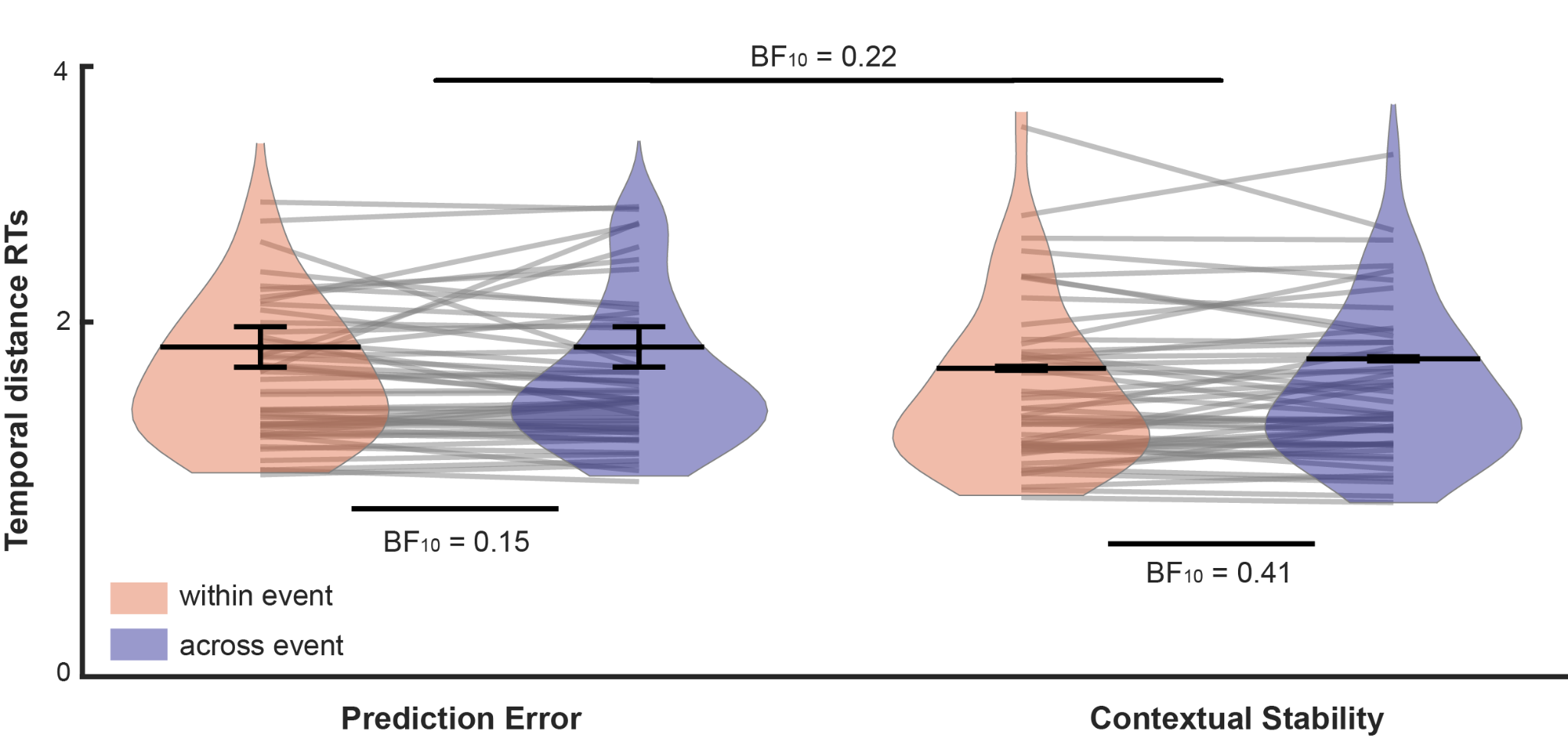


**Supplementary Figure 9.** Temporal distance judgments RTs for prediction error and contextual stability blocks in Experiment 3. The Bayes Factor above reflects the comparison of the segmentation scores (within-across difference) across conditions.


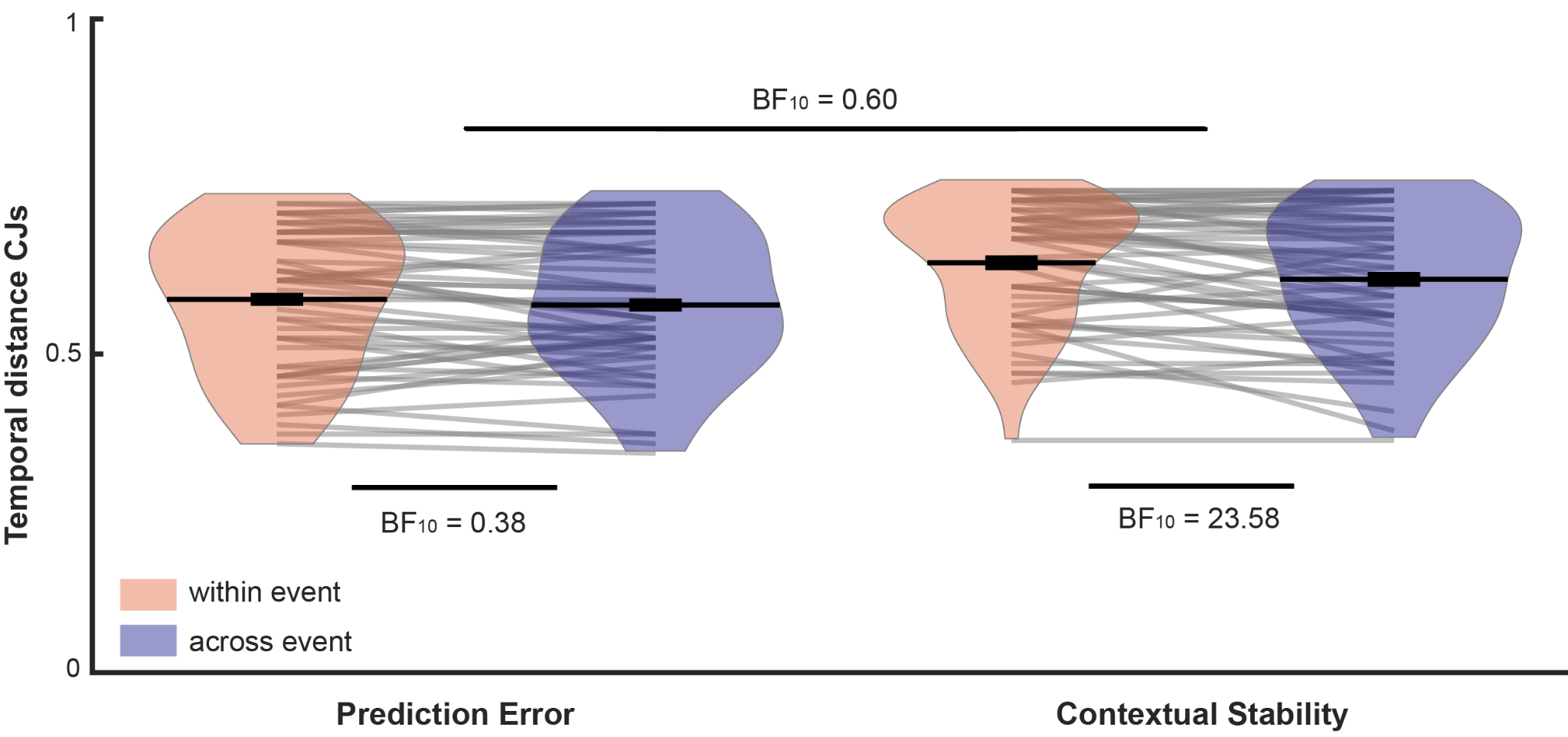


**Supplementary Figure 10.** Confidence judgments for temporal distance judgments for prediction error and contextual stability blocks in Experiment 3. The Bayes Factor above reflects the comparison of the segmentation scores (within-across difference) across conditions.


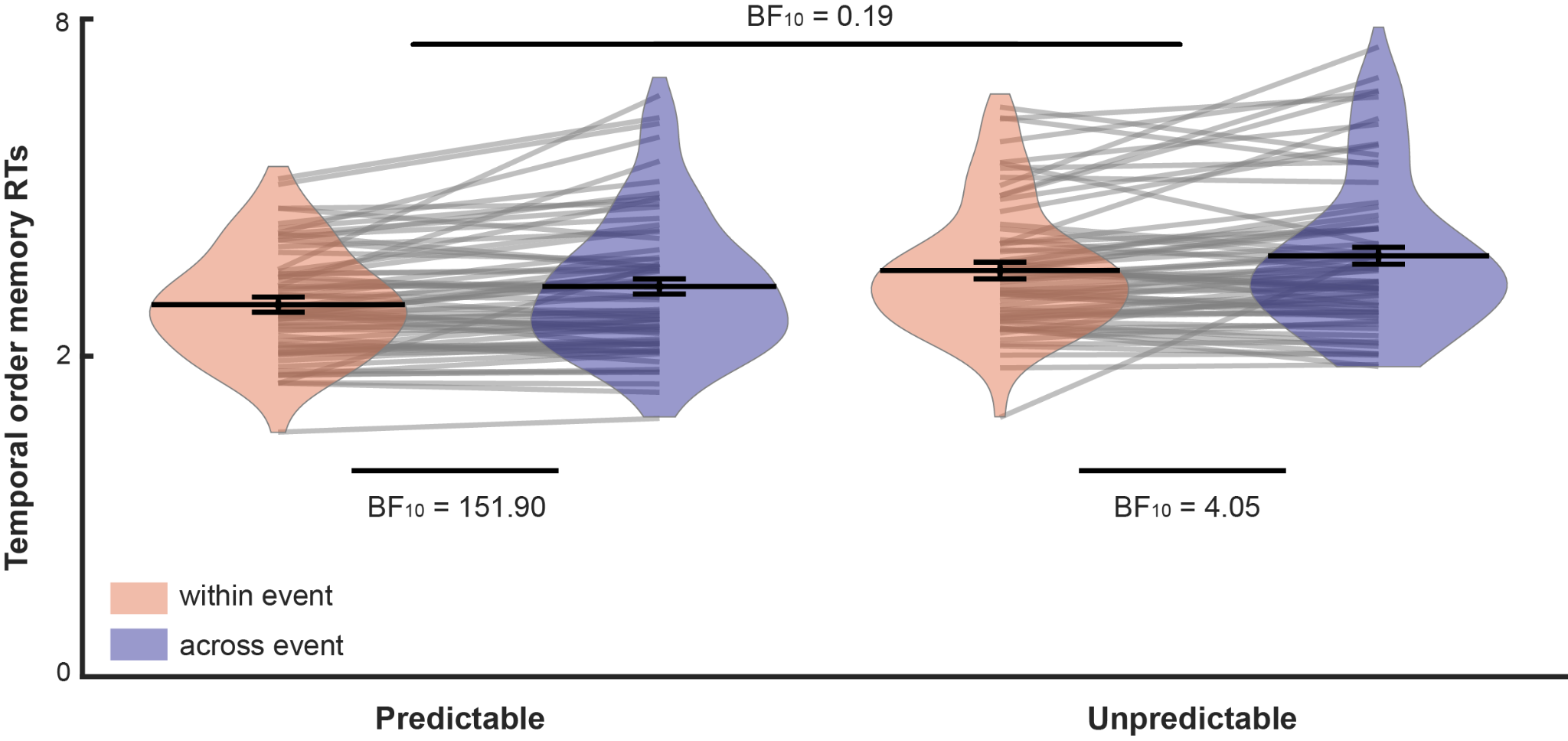


**Supplementary Figure 11.** Temporal order memory RTs for predictable and unpredictable blocks in Experiment 4. The Bayes Factor above reflects the comparison of the segmentation scores (within-across difference) across conditions.


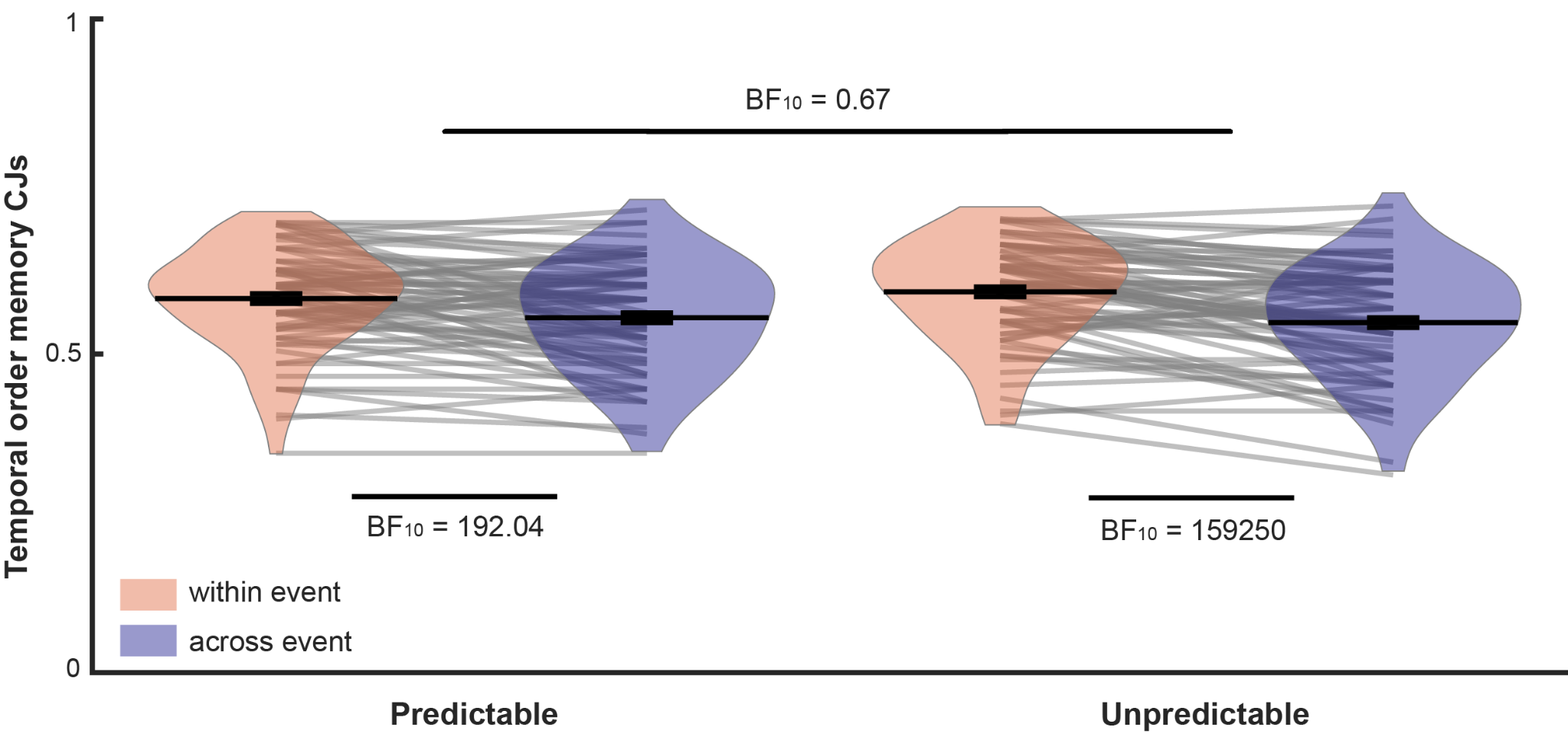


**Supplementary Figure 12.** Temporal order memory confidence judgments for predictable and unpredictable blocks in Experiment 4.The Bayes Factor above reflects the comparison of the segmentation scores (within-across difference) across conditions.


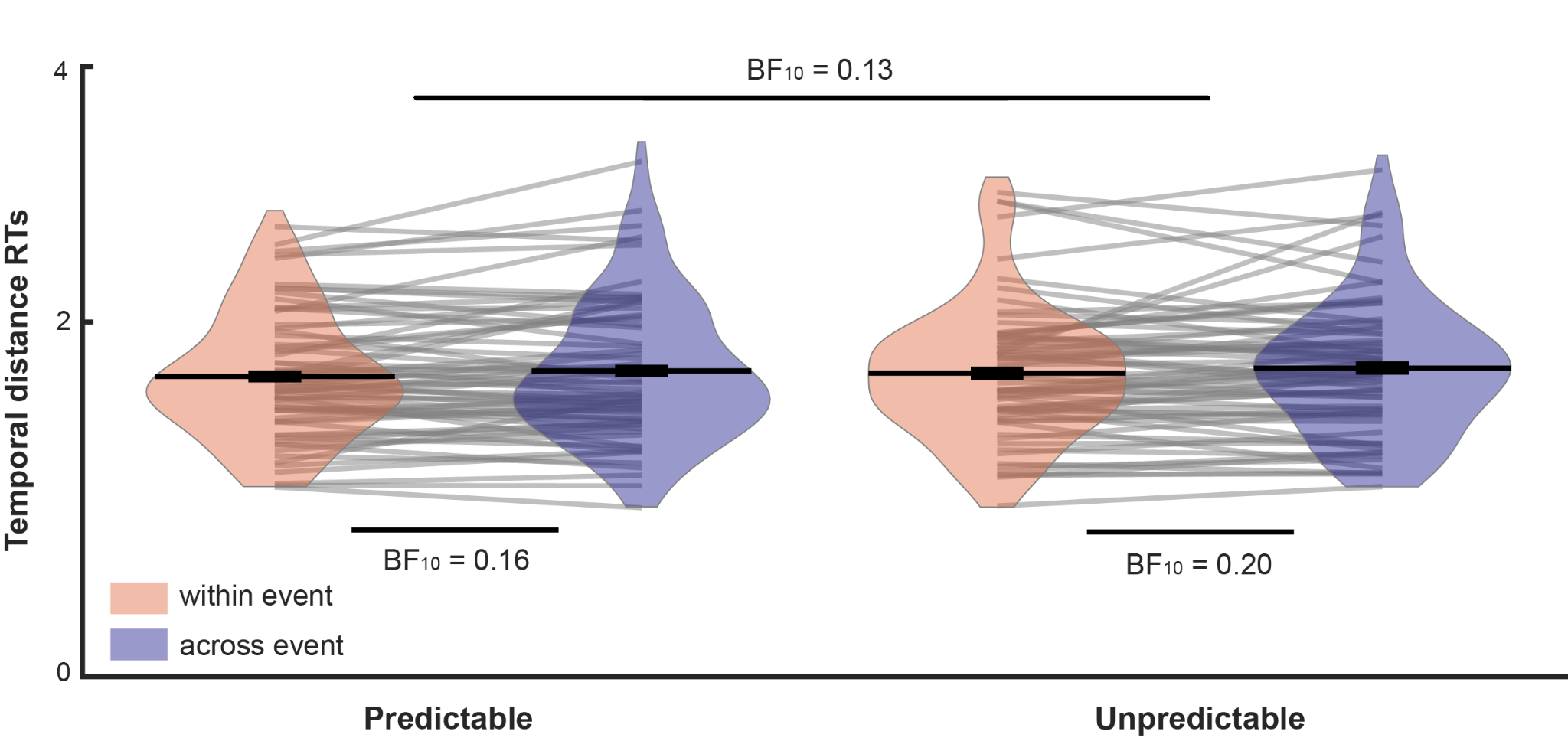


**Supplementary Figure 13.** Temporal distance judgments RTs for predictable and unpredictable blocks in Experiment 4. The Bayes Factor above reflects the comparison of the segmentation scores (within-across difference) across conditions.


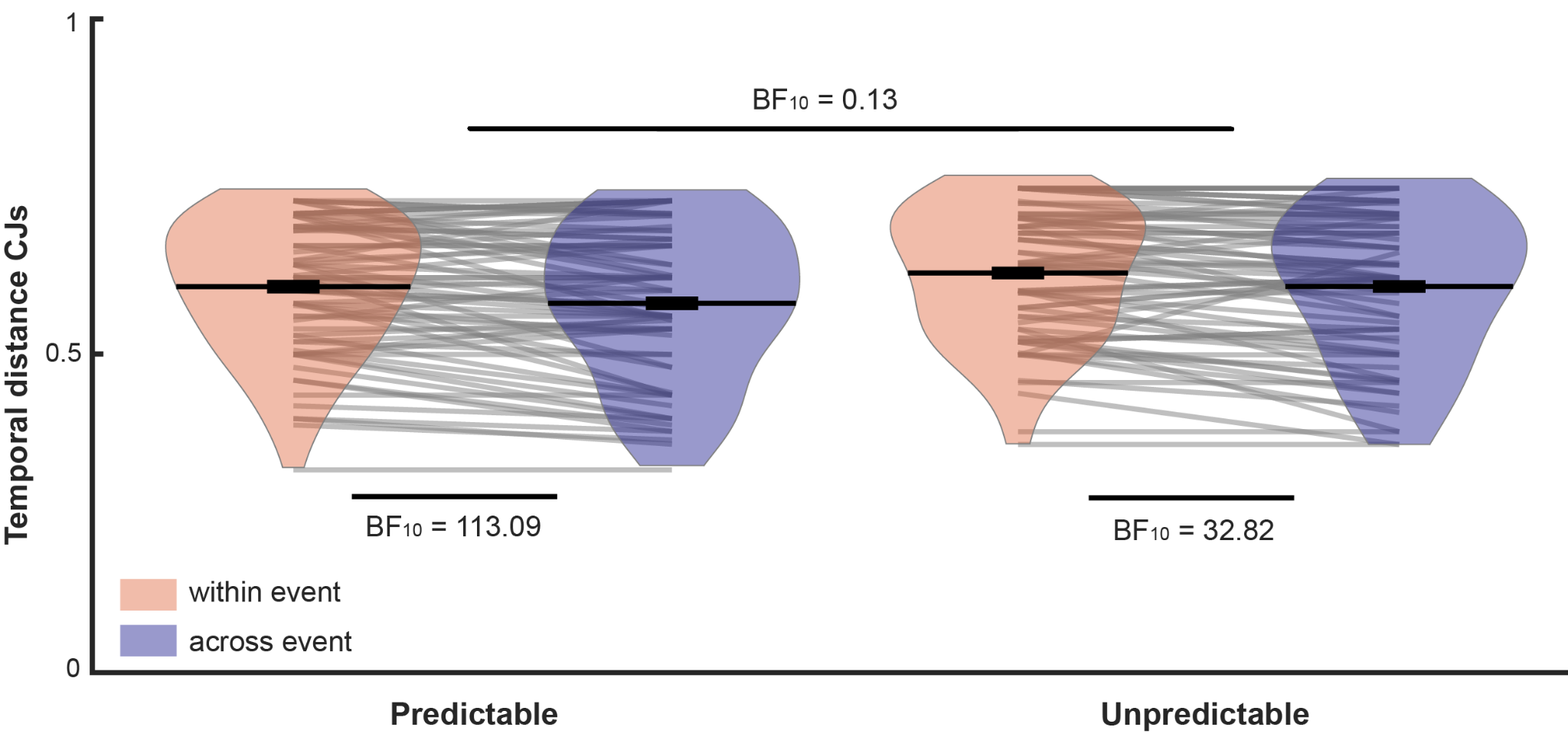


**Supplementary Figure 14.** Confidence judgments for temporal distance judgments for predictable and unpredictable blocks in Experiment 4. The Bayes Factor above reflects the comparison of the segmentation scores (within-across difference) across conditions.
